# Wildtype heterogeneity contributes to clonal variability in genome edited cells

**DOI:** 10.1038/s41598-022-22885-8

**Published:** 2022-10-28

**Authors:** Lukas Westermann, Yong Li, Burulca Göcmen, Matthias Niedermoser, Kilian Rhein, Johannes Jahn, Isabel Cascante, Felix Schöler, Niklas Moser, Björn Neubauer, Alexis Hofherr, Yvonne Lisa Behrens, Gudrun Göhring, Anna Köttgen, Michael Köttgen, Tilman Busch

**Affiliations:** 1 Renal Division, Department of Medicine, Medical Center, Faculty of Medicine, University of Freiburg, Freiburg, Germany; 2 Institute of Genetic Epidemiology, Faculty of Medicine and Medical Center, University of Freiburg, Freiburg, Germany; 3 CIBSS - Centre for Integrative Biological Signalling Studies, Freiburg, Germany; 4 Department of Human Genetics, Hannover Medical School, Hannover, Germany

**Keywords:** Cell biology, Nephrology

## Abstract

Genome editing tools such as CRISPR/Cas9 enable the rapid and precise manipulation of genomes. CRISPR-based genome editing has greatly simplified the study of gene function in cell lines, but its widespread use has also highlighted challenges of reproducibility. Phenotypic variability among different knockout clones of the same gene is a common problem confounding the establishment of robust genotype–phenotype correlations. Optimized genome editing protocols to enhance reproducibility include measures to reduce off-target effects. However, even if current state-of-the-art protocols are applied phenotypic variability is frequently observed. Here we identify heterogeneity of wild-type cells as an important and often neglected confounding factor in genome-editing experiments. We demonstrate that isolation of individual wild-type clones from an apparently homogenous stable cell line uncovers significant phenotypic differences between clones. Strikingly, we observe hundreds of differentially regulated transcripts (477 up- and 306 downregulated) when comparing two populations of wild-type cells. Furthermore, we show a variety of cellular and biochemical alterations in different wild-type clones in a range that is commonly interpreted as biologically relevant in genome-edited cells. Heterogeneity of wild-type cells thus contributes to variability in genome-edited cells when these are generated through isolation of clones. We show that the generation of monoclonal isogenic wild-type cells prior to genomic manipulation reduces phenotypic variability. We therefore propose to generate matched isogenic control cells prior to genome editing to increase reproducibility.

## Introduction

In the last decade, genome editing tools such as CRISPR/Cas9 have transformed the ease and precision of site-specific genome modifications^1–3^. Among many other applications, these molecular tools enable efficient genome editing of somatic cell lines to study effects of loss- or gain-of-function alleles^4^. Cultured cell lines have since been widely used to study gene function and to develop cell-based models of human disease^5,6^. The increasing number of such studies has drawn attention to the difficulty of establishing robust genotype–phenotype correlations and reproducible results using genome-edited cell lines.

The general challenges of achieving reproducible results with cell culture experiments have been well documented^7^. A wide range of factors, among them growth conditions, passage number, genetic drift, undetected contaminations or differing cell line origins, can lead to phenotypic variability and influence reproducibility of results^8–11^. In genome-edited cells, off-target effects have been described as a cause of phenotypic variability among different knockout (KO) clones of the same gene^12^. Therefore, extensive efforts have been devoted to reducing off-target effects in genome-edited cells^13–16^. However, even if current state-of-the-art protocols for genome editing are employed, considerable phenotypic variability can be observed.

We have encountered this problem studying the genes causing Autosomal Dominant Polycystic Kidney Disease (ADPKD), the most common monogenic disorder resulting in kidney failure. Mutations in the genes *Polycystic kidney disease 1* (*PKD1)* and *Polycystic kidney disease 2* (*PKD2*) cause ADPKD. There is an unmet need for robust cell-based models for ADPKD to study pathogenic pathways and to identify drug targets through high-throughput screening. We therefore applied CRISPR/Cas9 to generate KO cells of *Pkd1* and *Pkd2* in different well-established kidney epithelial cell lines^17^. To reduce variability at the gene level, we generated null alleles of both genes by deleting the entire coding region. This eliminates uncertainty about putative residual protein function, which may confound the analysis of alleles harboring small indels^18^.

However, despite strictly adhering to current protocols for cell culture and genome editing, we still observed considerable variability of phenotypes when analyzing multiple KO clones of the same genes. We therefore decided to investigate additional factors contributing to clonal variability. After transfection of CRISPR constructs, standard protocols for the generation of stable genome-edited cell lines usually require clonal isolation of a successfully genome-edited monoclonal cell out of a pool of wild-type (WT) cells^19^. These parental WT cells are typically used as experimental controls, assuming genetically and phenotypically homogeneous WT cell populations. We hypothesized that heterogeneity of WT cells might contribute to the observed phenotypic variability in genome-edited cells.

Here we test this hypothesis by studying the knockout of the *Pkd1* gene in stable kidney epithelial cells as a proof of concept. We demonstrate that WT cells display considerable clonal variability that contributes to phenotypic variability of genome-edited cells. Moreover, we show that the generation of isogenic WT subclones prior to genome editing reduces clonal heterogeneity of genome-edited cells.

## Results

### Genome-edited *Pkd1*-deficient cells show clonal variability

**Figure 1:**
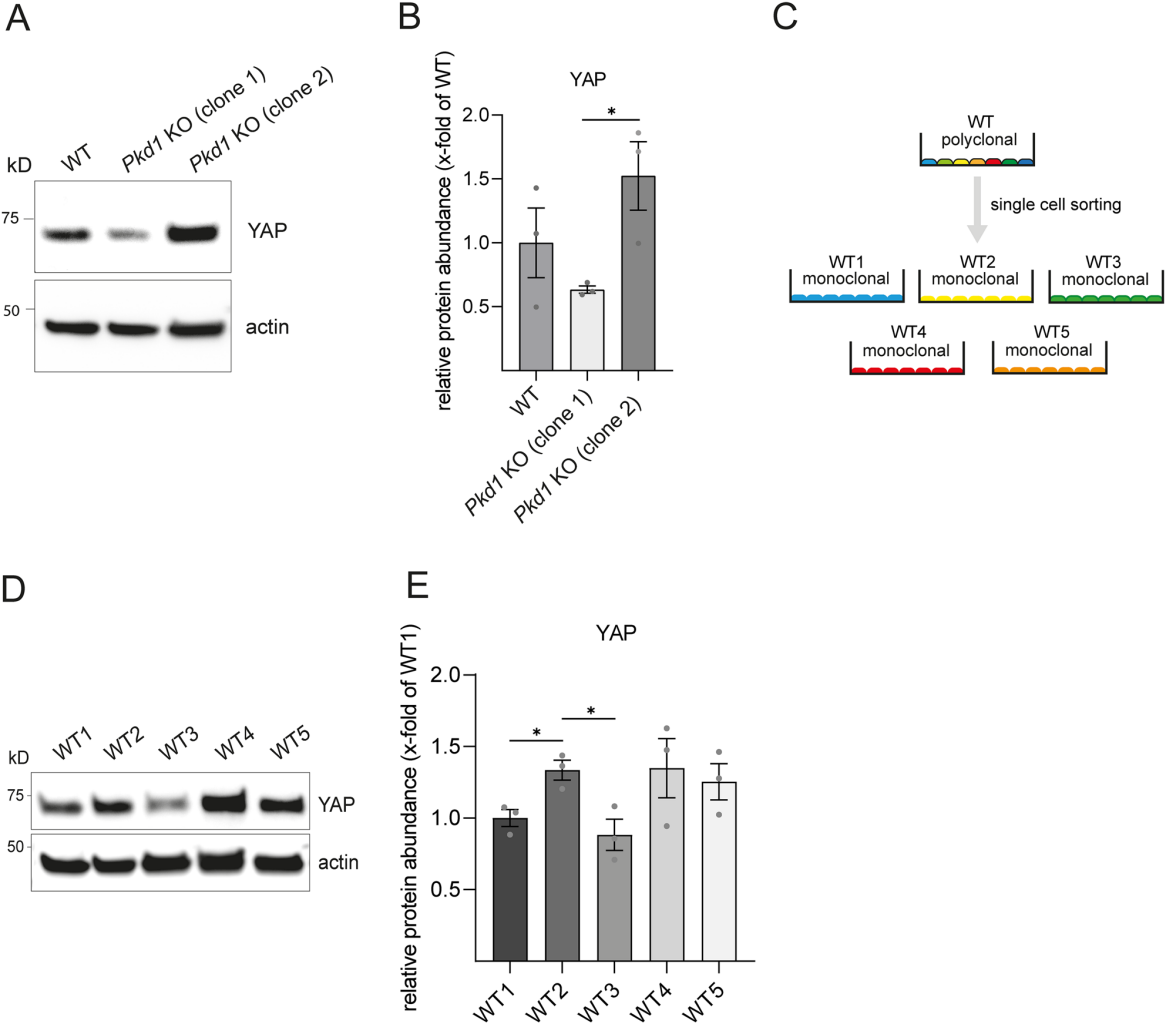
mIMCD-3 *Pkd1* knockout clones and monoclonal mIMCD-3 wild-type clones vary in protein abundance of YAP (**A**) Protein abundance of YAP was analyzed in mIMCD-3 WT cells compared to two different *Pkd1* KO clones after western blotting. (**B**) Densitometry shows significant differences in protein abundance for YAP in both *Pkd1* KO clones. (**C**) WT cells were single cell sorted by FACS generating monoclonal WT cells. Five different monoclonal cell populations were arbitrarily labeled WT1–WT5. (**D**) Protein abundance of YAP was analyzed in monoclonal mIMCD-3 WT cells. (**E**) Densitometry shows varying levels of YAP with significant differences between WT1 and WT2, and WT2 and WT3, respectively. Three independent experiments were included in analysis. Error bars represent SEM. Statistical significance was evaluated using unpaired t-test. *Indicates p < 0.05. Unprocessed blots for (**A** and **D**) are presented in Supplementary Figs. S6 and S7.

*PKD1* encodes Polycystin-1 (PC-1), a transmembrane protein that has been implicated in the regulation of multiple signaling pathways. Recently, the Hippo pathway effector protein YAP has been identified as a downstream target of PC-1 and modulator of polycystic phenotype severity in murine ADPKD models^20^. Protein levels of YAP were shown to be upregulated in human and murine *Pkd1*-deficient kidneys. Since kidney cysts in ADPKD arise from tubular epithelial cells^21^, we analyzed YAP levels in a murine *Pkd1-*deficient kidney epithelial cell line (mIMCD-3) we had previously established^17^. Surprisingly, YAP levels were decreased in *Pkd1*-deficient cells (clone 1) compared to WT control in contrast to the published data (Fig. 1A). To verify the observed YAP reduction, we analyzed a second *Pkd1*-deficient clone (clone 2) generated in the same genome editing experiment. Yet, YAP levels of the second *Pkd1* KO clone were increased compared to WT control and significantly higher compared to *Pkd1* KO clone 1 (Fig. 1A,B).

One possible and often cited explanation for the observed clonal variability could be the occurrence of CRISPR/Cas9-related off-target events^12^. However, we had taken measures to reduce off-target events including the use of gRNAs with low off-target prediction as well as a Cas9 nickase. In addition, we also observed clonal heterogeneity in several TALEN-generated KO cell lines (data not shown) which are thought to have a lower probability of off-target effects^22^. Furthermore, all *Pkd1* KO clones used in this study are confirmed null alleles. Thus allelic variants with different functional effects (gain*-* versus loss-of-function) and severity (hypomorphic versus complete loss-of-function) can be ruled out. Together this suggested that other factors may contribute to variable results in clonal genome-edited cells. We therefore hypothesized that phenotypic variability in genome-edited cells can be caused by intrinsic heterogeneity of the WT cells from which they arise.

### Monoclonal wild-type mIMCD-3 cells display significant phenotypic variability

To address the potential influence of WT heterogeneity, we decided to perform single cell sorting of mIMCD-3 WT cells in order to generate monoclonal WT cell lines (Fig. 1C). We first screened monoclonal WT cells for the presence of primary cilia since ADPKD is a so-called ciliopathy—a group of disorders characterized by structural or functional defects of primary cilia^23^. Consequently, a cell-based model for ADPKD should incorporate cilia. Surprisingly, immunofluorescence staining for acetylated tubulin as a ciliary marker did not show any signal in some clones (data not shown). For further characterization, we only selected cilia-positive mIMCD-3 cell clones which we labeled WT 1–5. These clones were analyzed for protein abundance of YAP (Fig. 1D). Quantitative analysis showed significant alterations in YAP levels among these clones (Fig. 1E). Notably, the magnitude of differences was comparable to those in genome-edited cells (Fig. 1A). Moreover, alterations of protein levels in different wild-type clones were not restricted to YAP. We also conducted experiments with a set of kinases (pPERK, CaMKII delta and pAMPK) which have been shown to be linked to *Pkd* signaling^24–26^. Quantitative analysis of protein expression demonstrated significant alterations in protein levels for these kinases among monoclonal WT clones (Supplementary Fig. S1). In summary, substantial differences in protein levels were found between WT clones in a range which is often interpreted as biologically relevant.

To test whether clonal variability also manifests in other assays, we investigated cellular survival in the presence of cytotoxic compounds. Drug susceptibility was interrogated by generating dose–response curves for survival with three monoclonal and the parental polyclonal WT cell lines. Dose–response curves were generated for two commonly used nephrotoxic drugs, cisplatin and methotrexate. Drug susceptibility for cisplatin was not significantly altered between WT clones (Supplementary Fig. S2A,B). In contrast, treatment with the dihydrofolate reductase inhibitor methotrexate led to reproducible and significant changes in viability of multiple WT clones (Supplementary Fig. S2C,D).

**Figure 2:**
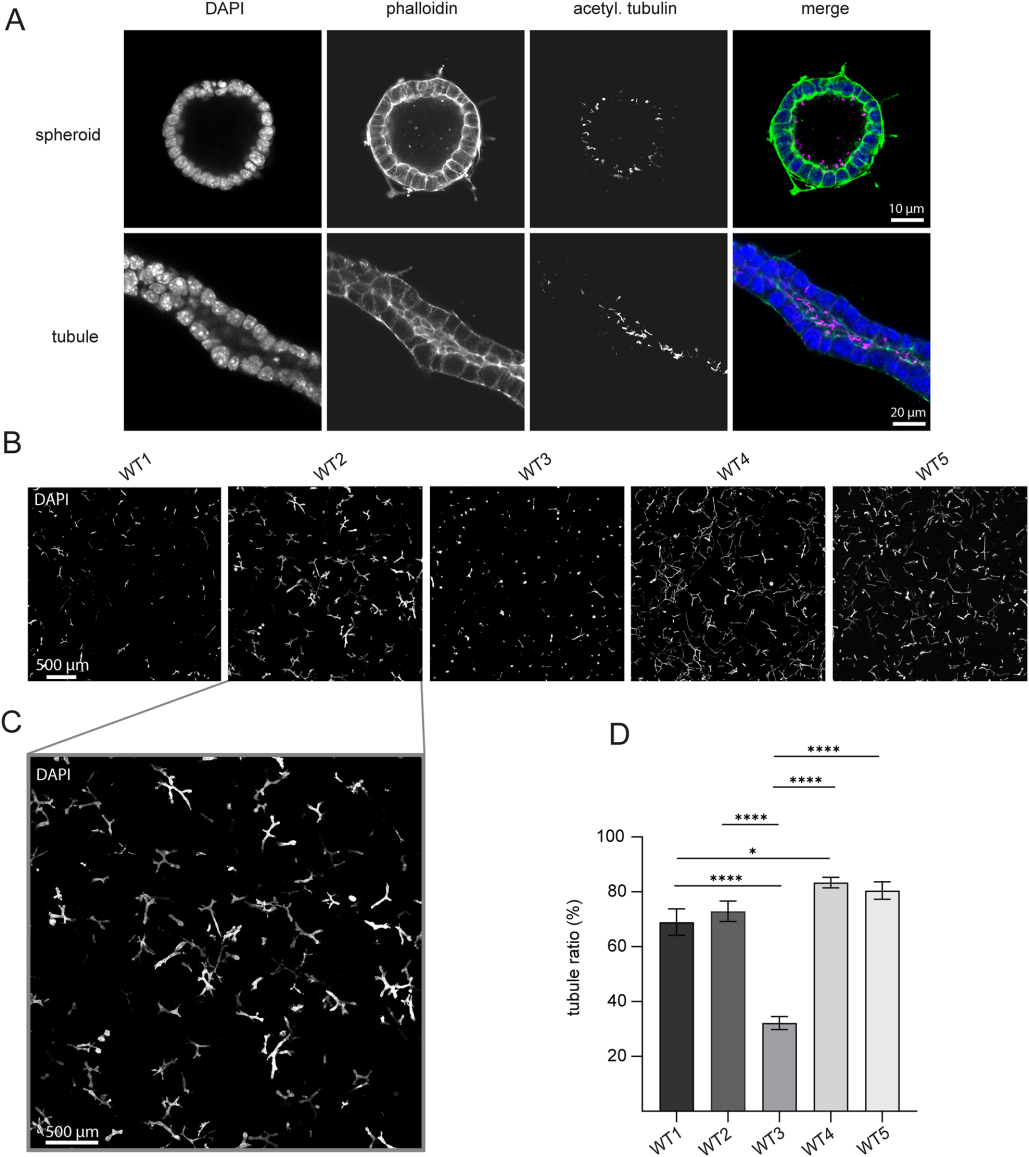
Monoclonal mIMCD-3 wild-type cells show different morphology in 3D cell culture (**A**) Predominant structures of a kidney tubule organoid assay derived from mIMCD-3 WT cells. Representative spheroid (upper row) and representative kidney tubule (lower row) in a 3D-Matrigel-collagen scaffold. Images were acquired with confocal microscopy. (**B**) Representative images of five different monoclonal mIMCD-3 WT cells in a 3D-Matrigel-collagen scaffold. WT clones show different growth patterns, e.g. induction of kidney tubules is highly impaired in WT3 compared to other clones. (**C**) Magnification of WT2-image from panel B illustrates high number of differentiated kidney tubules in this clone. (**D**) Quantification of tubule ratio (tubules/ all structures) of five monoclonal mIMCD-3 WT clones. Tubule ratio varies significantly between clones. Bars represent mean tubule ratio of three independent experiments (four technical replicates each). Error bars represent SEM. Statistical significance was evaluated using unpaired t-test.*Indicates p < 0.05. ****Indicates p < 0.0001.

To study phenotypic variability in more complex biological programs, we analyzed tubulogenesis in 3D cell cultures. 3D cell cultures are increasingly used for disease modelling and dissection of mechanistic biological processes^27^. We and others use kidney tubule organoids to investigate Polycystin signal transduction and the mechanisms of tubulogenesis in vitro. Predominant structures of our 3D cell culture model can be divided into cyst-like epithelial structures (spheroids*)* and tubular epithelial structures (tubules*)* (Fig. 2A). Screening of monoclonal mIMCD-3 WT clones for their tubulogenic potential and proliferative features revealed significant phenotypic differences between distinct mIMCD-3 WT clones (Fig. 2B–D). Consequently, dissecting gene function in 3D cell cultures with polyclonal cell lines can be biased by heterogeneity of WT cells resulting in misinterpretation of gene deletion effects after isolation of subclones.

In summary, different WT subclones derived from the same well-characterized mIMCD-3 cell line show remarkably different characteristics regarding abundance of proteins, drug susceptibility and morphological patterns in 3D cell cultures. We thus concluded that WT heterogeneity might be a driver for phenotypic variability of genome-edited cell lines. This led us to modify our genome editing protocol and introduce an initial single cell sorting step to generate monoclonal WT cell lines prior to transfection of gRNAs and Cas9 (Supplementary Fig. S3).

### KO clones derived from monoclonal isogenic wild-type cells show less variability and differences in protein levels compared to polyclonal KO clones

**Figure 3:**
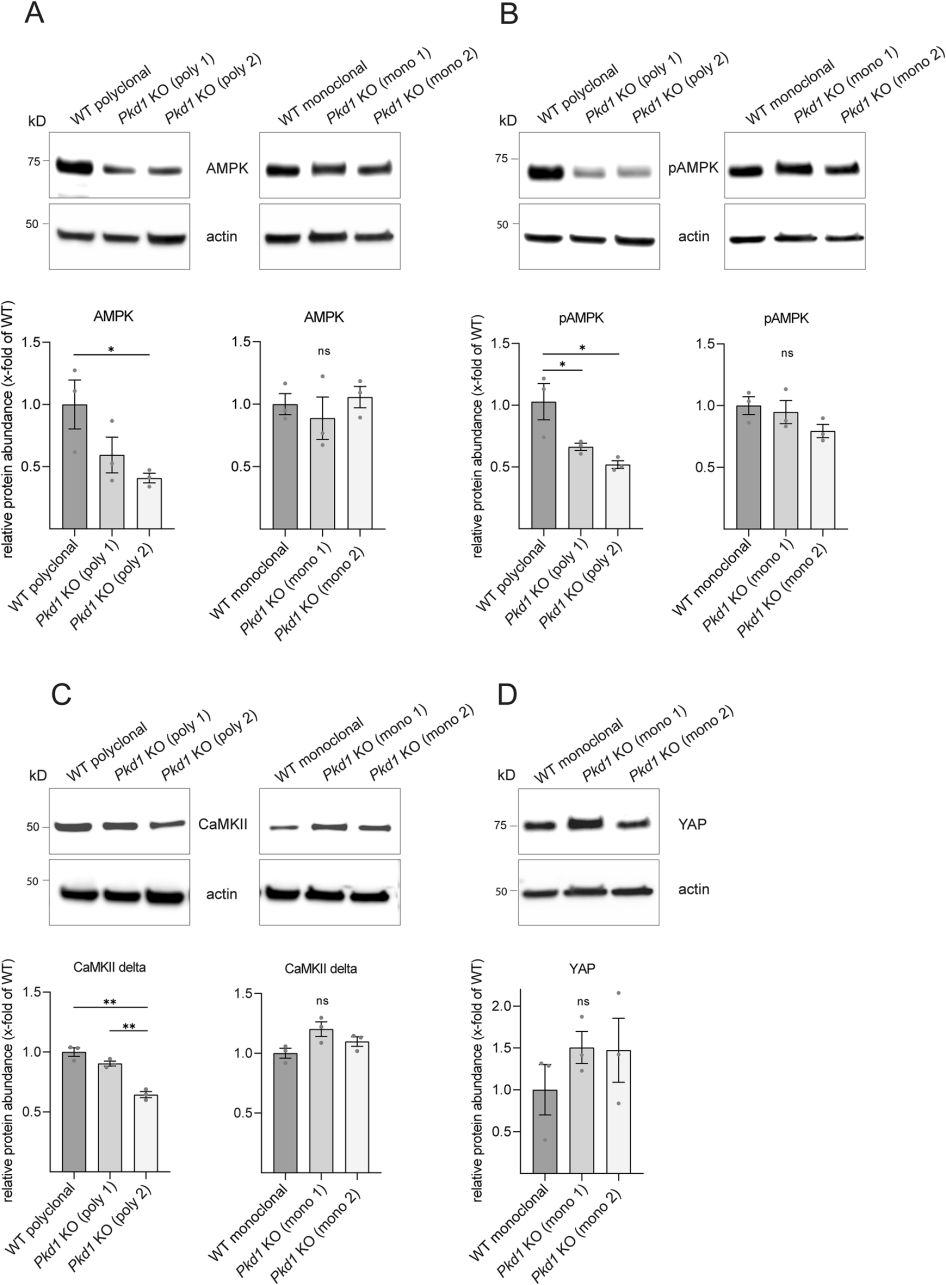
Variability of protein levels for AMPK, pAMPK, CaMKII delta and YAP is reduced in monoclonal compared to polyclonal *Pkd1* knockout clones (**A**) Protein abundance of AMPK is significantly reduced in *Pkd1* KO clone 2 compared to polyclonal WT control. Monoclonal *Pkd1* KO clones show no significant differences in protein abundance of AMPK compared to monoclonal WT control. (**B**) Protein abundance of pAMPK is significantly reduced in both polyclonal *Pkd1* KO clones compared to polyclonal WT control. Monoclonal *Pkd1* KO clones show no significant differences compared to isogenic WT control. (**C**) Protein abundance of CaMKII delta is significantly reduced in *Pkd1* KO clone 2 compared to polyclonal WT control and *Pkd1* KO clone 1. Monoclonal *Pkd1* KO clones show no significant differences compared to isogenic WT control. (**D**) Monoclonal *Pkd1* KO clones show no significant differences in protein abundance of YAP compared to isogenic WT control. Polyclonal setting is illustrated in Fig. 1. Three independent experiments were included in each analysis of protein levels. Error bars represent SEM. Statistical significance was evaluated using unpaired t-test. *Indicates p < 0.05. **Indicates p < 0.01. ***Indicates p < 0.001. ns means non-significant. Unprocessed blots for (**A**–**D**) are presented in Supplementary Figs. S8, S9, S10, and S11.

Using this modified genome editing workflow, we generated monoclonal *Pkd1* KO clones from a matched isogenic WT control cell line. In the following, we refer to these cells as monoclonal *Pkd1* KO cells. Cell lines generated from polyclonal parental cells are termed polyclonal KO cells. Again, we analyzed expression levels of polycystic kidney disease signaling related kinases in these new monoclonal *Pkd1* KO cell lines and compared this to results obtained in polyclonal KO cell lines^24,25^. This comparison showed significant differences: while polyclonal *Pkd1* KO clones showed a significant reduction of 5' adenosine monophosphate-activated protein kinase (AMPK) compared to polyclonal WT control, monoclonal *Pkd1* KO clones showed no significant change in AMPK levels compared to an isogenic WT control (Fig. 3A). Similarly, levels of phosphorylated monophosphate-activated protein kinase (pAMPK) were significantly changed in polyclonal *Pkd1* KO clones, but not in the monoclonal *Pkd1* KO clones (Fig. 3B). Notably, investigation of genotype-dependent changes of protein levels of CaMKII delta in polyclonal *Pkd1* KO clones showed variable results. Polyclonal *Pkd1* KO clone 1 showed identical CaMKII delta levels compared to the WT control (Fig. 3C). In contrast, *Pkd1* KO clone 2 showed a significant reduction compared to both WT and *Pkd1* KO clone 1. In the monoclonal setting, levels of CaMKII were not significantly different. Similarly, YAP levels of both monoclonal *Pkd1* KO clones were almost identical with a non-significant increase compared to WT control (Fig. 3D). In contrast, we observed a high degree of clonal variability with opposite results in polyclonal *Pkd1* KO clones (Fig. 1). In summary, experiments using monoclonal *Pkd1* KO cells with matched isogenic control cells led to reduced variability.

### Monoclonal and polyclonal wild-type cells show profound transcriptomic differences

**Figure 4:**
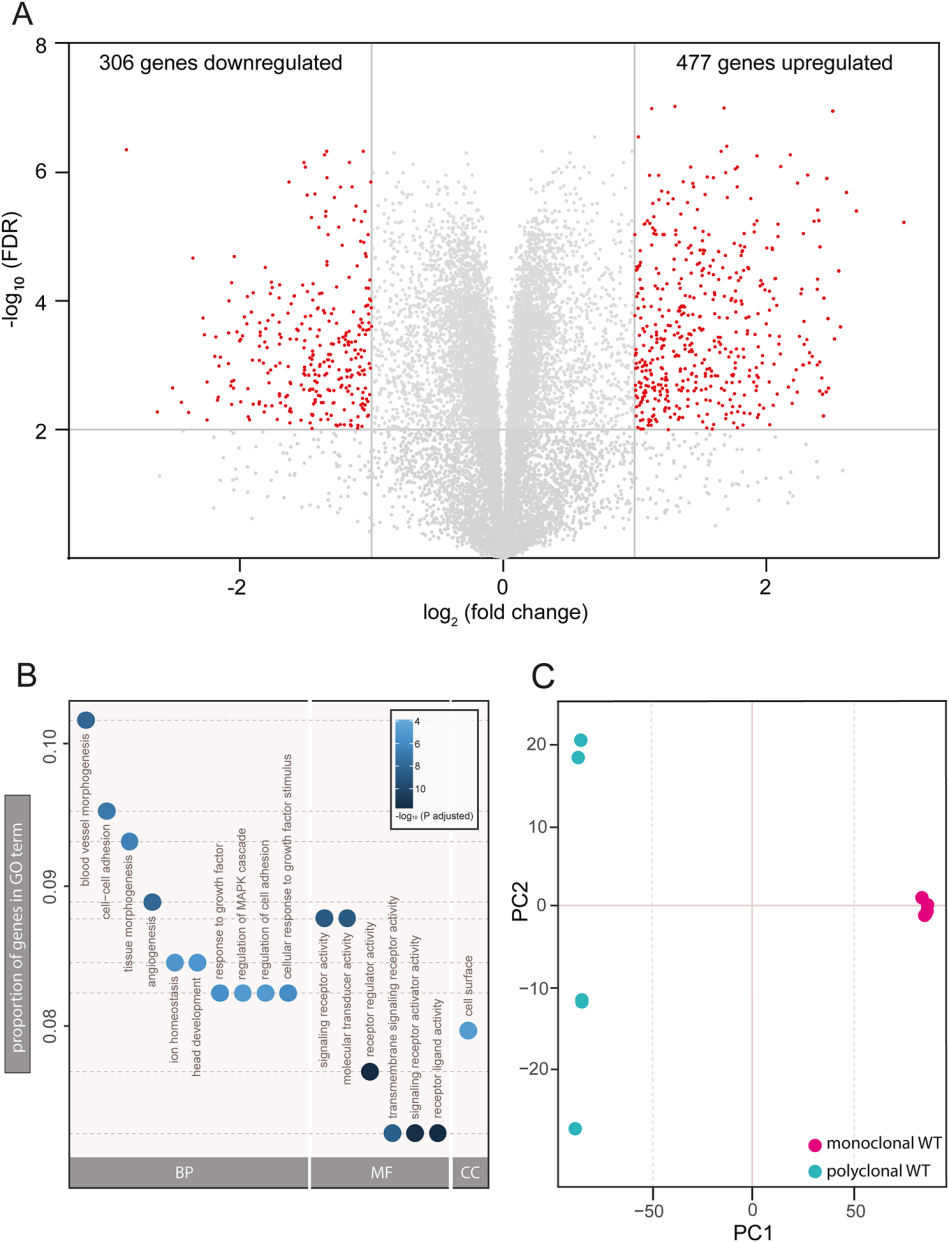
RNA sequencing analysis reveals altered expression levels between mono- and polyclonal wild-type cell lines and confirms heterogeneity of polyclonal cells. RNA was extracted from poly- and monoclonal (WT5) mIMCD-3 WT cell lines. RNA sequencing was performed for four technical replicates each. (**A**) Differential gene expression between poly- and monoclonal (WT5) cell lines are illustrated in a volcano plot. Threshold for false discovery rate (FDR) was set to log_10_ ≥ 2. Threshold for fold change was set to log_2_ ± 1. Grey dots represent genes which do not meet threshold criteria (FDR log_10_ < 2 and/or log_2_ (fold change) between ± 1). Red dots indicate significant changes in expression. 306 genes are significantly downregulated, 477 genes are significantly upregulated in monoclonal compared to polyclonal mIMCD-3 WT cell lines (adjusted p-value < 0.01). (**B**) Gene ontology (GO) term analysis shows most significant changes in expression levels of genes in dependence of gene ontology classification. Y-axis depicts proportion of genes in GO term analysis. Each dot represents distinct ontologies. Colour of dots represents significance of changes in expression levels between mono- and polyclonal WT-cells. Colour scheme depicts significance levels (− log_10_ p-adjusted). *BP* biological process, *MF* molecular function, *CC* cellular components. (**C**) Principal component (PC) analysis shows variability between sequencing replicates. Monoclonal sequencing replicates cluster together in contrast to polyclonal sequencing replicates showing a heterogeneous pattern.

To obtain a more global view on differences between monoclonal and polyclonal WT cells as a possible confounding factor in genome editing experiments, we performed an unbiased transcriptome-wide analysis using RNA sequencing. Differential gene expression analyses are frequently conducted to compare genome-edited with WT cell lines. They allow for the identification of genes or pathways revealing information about physiological or pathophysiological processes^28–30^. We compared the transcriptomic profiles of mono- and polyclonal WT cells to uncover the potential contribution of clonal isolation to differential gene expression. Strikingly, transcriptomic analysis of mono- vs polyclonal WT cells revealed a large number of differentially regulated genes despite stringent cut-off criteria (Fig. 4A; Supplementary Data 1). In monoclonal WT cells 477 genes were significantly upregulated while 306 genes were downregulated compared to polyclonal WT cells (adjusted p < 0.01). Likewise, Gene Ontology (GO) term analysis yielded a variety of significantly enriched GO terms when comparing mono- and polyclonal WT cells (Fig. 4B). Of note, the most significant altered GO terms between mono*-* and polyclonal cells are linked to receptor and signaling activity. Such a result within the context of an experiment comparing *Pkd1* KO and WT cells would easily be interpreted as meaningful, given the role of PC-1 as a transmembrane receptor. In fact, however, this significant enrichment was purely derived from comparing mono- and polyclonal WT cells. Finally, principal component analysis (PCA) confirmed that variability of biological replicates is drastically reduced when using monoclonal cell lines (Fig. 4C), a finding which is in line with our prior experiments. Taken together, these transcriptomic analyses identify WT heterogeneity as a significant confounding factor in experiments involving clonal isolation.

### Monoclonal cell line and its subclones show similar transcriptomic profiles

**Figure 5:**
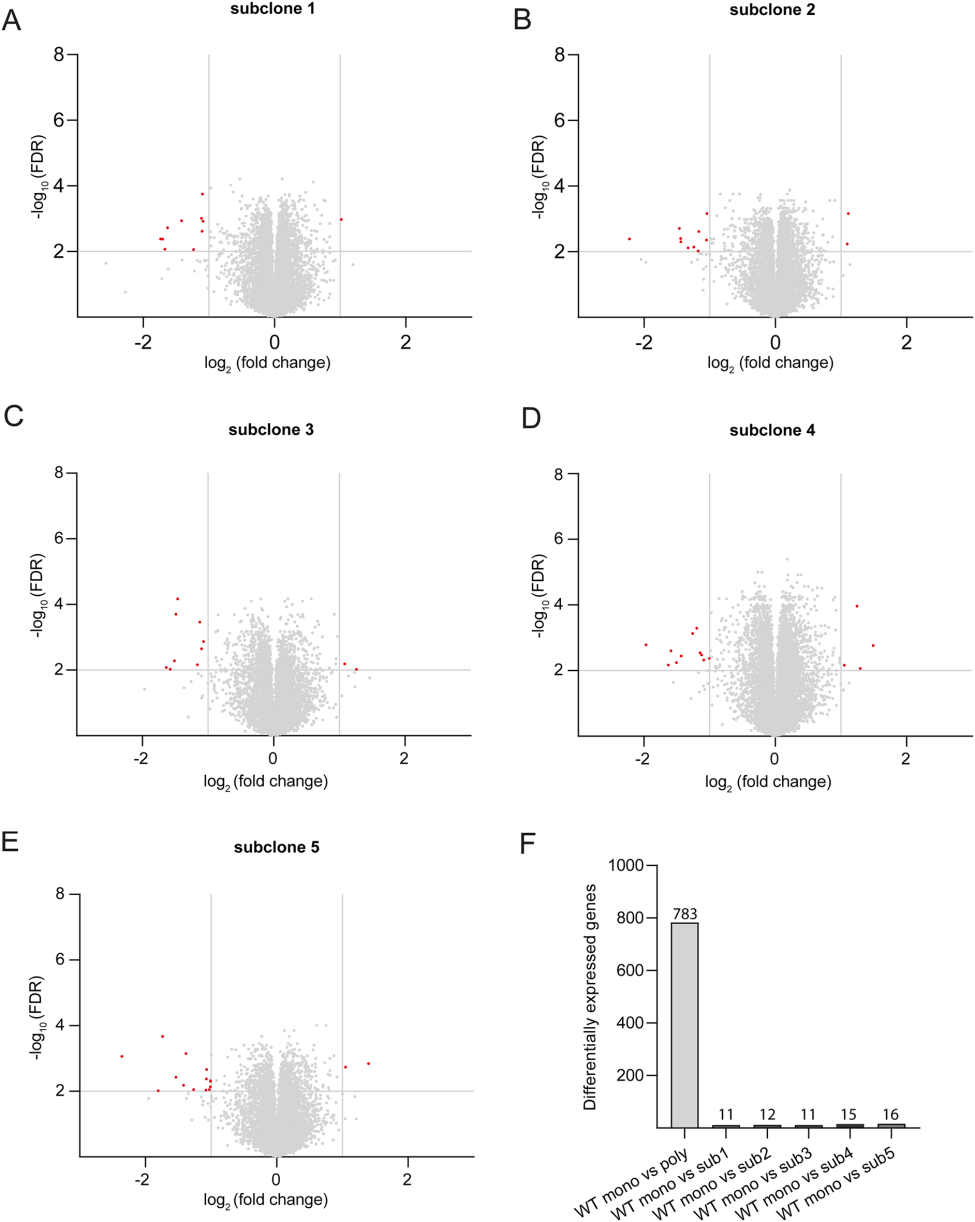
Monoclonal wild-type cell line and corresponding subclones show similar transcriptomic profiles in contrast to the polyclonal wild-type. Volcano plots with differentially expressed genes in five different subclone cell lines of the parental monoclonal cell line in comparison with the parental monoclonal cell line (**A**–**E**). A bar chart (**F**) summarizes total amount of differentially expressed genes comparing parental cell line with polyclonal wild-type cell line as well as subclones with parental cell line. Threshold for false discovery rate (FDR) was set to log_10_ ≥ 2. Threshold for fold change was set to log_2_ ± 1. Grey dots represent genes which do not meet threshold criteria (FDR log_10_ < 2 and/or log_2_ (fold change) between ± 1).

In a transcriptomic comparison of mono- and polyclonal wild-type cell lines we observed hundreds of differentially regulated genes (Fig. 4A). In western blot experiments we showed less variability in experiments involving monoclonal genome-edited cells and its corresponding control cell line compared to polyclonal cell lines. Based on these observations, we hypothesized that subclones derived from a monoclonal isogenic cell line should be more similar to their parental cell line than those derived from polyclonal cells. To test this hypothesis, we performed single cell sorting of WT clone 5 and expanded five resulting subclones over a period of approximately 6 weeks. After this timespan we isolated RNA of these subclones and performed RNA sequencing. Notably, all investigated subclones and their parental cell line had highly similar transcriptomic profiles (Fig. 5A–E; Supplementary Data 2) with a low amount of differentially expressed genes (mean differentially expressed genes: 13). In contrast, the transcriptomic comparison between monoclonal WT5 and the polyclonal WT cell line resulted in 783 differentially expressed genes (Fig. 5F). Therefore, the amount of differentially expressed genes is drastically reduced by using subclones generated from a monoclonal cell line.

### Phenotypic variability of monoclonal cell lines is not explained by chromosomal aberrations

Our results show that mIMCD-3 cells are polyclonal in nature and show distinct phenotypic characteristics. However, the underlying mechanism is unclear. Immortalized cell lines have been shown to be polyploid^31,32^. Polyploidy can result in genomic instability leading to structural aberrations^33–35^. Chromosomal instabilities could potentially explain the variation in phenotypes between monoclonal cell lines. We therefore asked if chromosomal aberrations are found in the monoclonal wild-type cell lines as well as in the *Pkd1* KO cell lines. To address this question, we conducted multicolor fluorescence in situ hybridizations for these cell lines. A comparison of karyograms between monoclonal wild-type cells and between polyclonal and monoclonal *Pkd1 KO* cell lines showed similar karyograms without evidence for structural aberrations or instabilities (Supplementary Fig. S4). Of note, our analysis did not reveal an increase in polyploidy in *Pkd1* KO clones as demonstrated in a previous study^36^. In conclusion, karyotype differences or chromosomal aberrations are probably not the cause for the observed phenotypic variability.

### Wild-type heterogeneity is a cause for clonal variability in genome editing experiments

**Figure 6:**
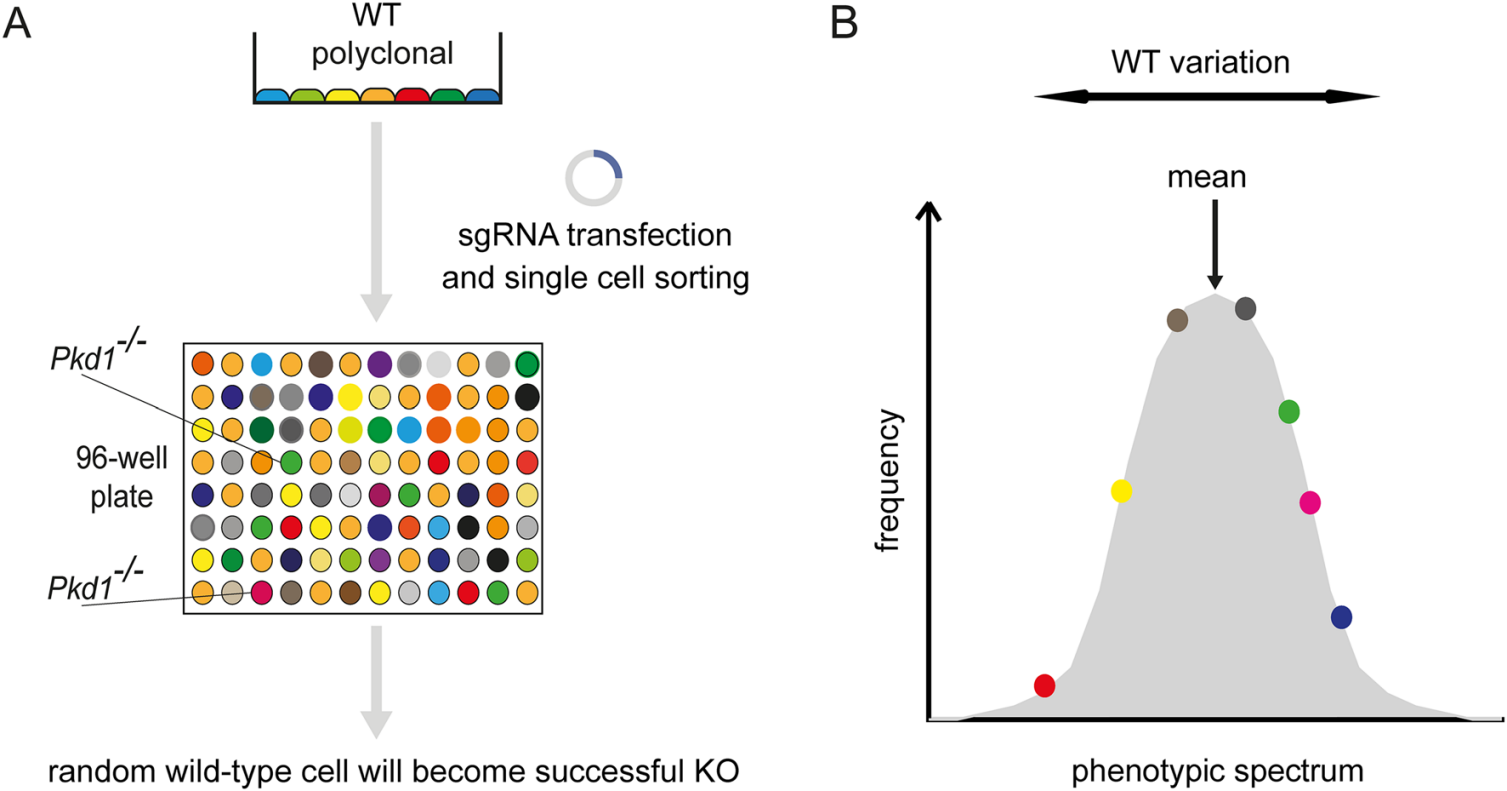
Model explaining heterogeneity of polyclonal knockout cell lines. (**A**) Our data shows that mIMCD-3 cells are polyclonal in nature. Genome editing results in KO cells derived from a random WT cell. Subsequent experiments are conducted with a heterogenic WT control, therefore confounding the analysis of gene deletion effects. (**B**) Polyclonal mIMCD-3 cells represent the mean of a phenotypic spectrum. Our data shows that monoclonal WT cells are heterogeneous concerning distinct phenotypes. In experiments, KO cells derived from random WT cells are compared with polyclonal, heterogenic WT cells. This can lead to misinterpretation of findings, since the observed phenotype may result from heterogeneity of WT cells instead of gene deletion.

In conclusion, our data show that mIMCD-3 WT cells are polyclonal and heterogeneous despite their monoclonal origin^37^, possibly through genetic drift. Hence, the isolation of subclones from polyclonal cells after genomic manipulation leads to propagation of cells with distinct features (Fig. 6A). In the typical setting of such experiments, a random WT cell with distinct features is converted into a successful KO clone. This has two problematic implications: (i) KO clones will display phenotypic variability due to their polyclonal origin and (ii) KO clones lack proper controls. Since polyclonal WT control cells represent the mean for an investigated phenotype, phenotypic alterations observed in subsequent experiments with KO clones can originate from deviations of a specific WT clone from the mean instead of being a consequence of genetic loss-of-function (Fig. 6B). Our findings identify heterogeneity of WT cells as a major confounding factor in genome editing experiments involving clonal isolation and show that the establishment of matched isogenic control cells increases reproducibility and reduces erroneous results.

## Discussion

Genome editing tools such as CRISPR/Cas9 have given cell biologists the opportunity to study genetic alterations such as loss- or gain-of-function mutations in cell culture models. Validity and reproducibility of results in cell culture models depend on strict adherence to an optimized experimental setting. Effect sizes are often small and hard to detect. Hence, to precisely dissect genotype–phenotype relationships, it is critical to minimize confounding factors.

Here, we study a frequently neglected confounding factor when using genome-edited cells. We demonstrate that the polyclonal nature of WT cells contributes to phenotypic variability in genome-edited cells. Cultured immortalized cell lines such as HeLa, CHO, HEK293, MDCK, or mIMCD-3 cells are extensively used for genome editing experiments^38–42^. They are well characterized, easy to propagate, and are considered to be rather stable phenotypically compared to primary cells. Therefore, these cell lines are suitable for many research applications including disease modeling and high-throughput screening for drug discovery. Some of these cell lines exhibit physiological characteristics of the tissue of origin. For example, mIMCD-3 cells used in this study retain many differentiated characteristics of the kidney collecting duct including apico-basal polarity, high transepithelial resistance and electrolyte transport^37^. Furthermore, mIMCD-3 cells are ciliated and therefore extensively used to study ciliopathies such as ADPKD^43–45^. Although mIMCD-3 cells were established as a monoclonal cell line, we now show that they are inhomogeneous and subclones feature a broad phenotypic spectrum regarding multiple traits. For instance, differential gene expression analysis of monoclonal vs. polyclonal WT cell lines leads to the detection of a large number of differentially expressed genes. After genome editing and subsequent single cell sorting, this phenotypic spectrum inherent to polyclonal WT cells can be misinterpreted as a consequence of CRISPR-induced genetic alterations. Of note, the issue of WT heterogeneity is likely further aggravated in cell types with higher inherent genetic and phenotypic variability such as reprogrammed human induced pluripotent stem cells, which are frequently used for genome editing experiments.

We further show that the confounding potential of heterogeneous WT cell lines can be reduced by establishing monoclonal cell lines by single cell sorting before genome editing. Our study demonstrates that this optimized workflow reduces (i) phenotypic variability of genome-edited cell lines as well as (ii) false-positive detection of genotype-dependent phenotypes. In principle, false-negative results are also possible, e.g. when the genetic manipulation results in a phenotype that is masked by an opposite phenotype from the WT spectrum of the selected genome-edited clone.

In addition to studying multiple genome-edited clones, rescue experiments or reversal of genome-editing can help to validate positive findings and eliminate false-positive results. This includes erroneous phenotypes due to variability of WT cell lines, but also those due to off-target effects of the genome editing process. Using monoclonal cell lines for genome editing has the potential to significantly minimize the time spent trying to verify results that ultimately prove not reproducible. Besides that, this approach might be useful in cases when rescue experiments are not feasible, e.g. when a narrow control of expression levels of a protein is necessary and technically not achievable or when investigating dominant negative mutations. We therefore propose to generate matched isogenic control cells prior to genome editing experiments involving isolation of clones. While this requires additional time, it will likely improve validity and reproducibility of such experiments.

When using monoclonal cell lines it should be considered that a single clone might not be representative for the totality of the cell line. Subsequently, generation and editing of several distinct clones should be considered. Furthermore, a specific clone might not be suited for the planned investigations, e.g. we encountered mIMCD-3 clones that did not develop primary cilia. Obviously, such a clone is not suited for further investigation of ciliopathies. Hence, phenotypic assessment of WT clones used for genome editing regarding desired features for further investigation is advised.

Although we demonstrated considerable phenotypic differences between monoclonal wild-type cell lines, the underlying mechanism for heterogeneity remains elusive. Karyogram analyses showed polyploidy for all investigated cell lines. However, structural aberrations were not observed. Genetic drift in cell lines due to spontaneous mutations has been frequently reported and does not necessarily require chromosomal aberrations^46^. It is therefore prudent to expand and freeze a high number of cells after clonal isolation as suggested in our protocol (Supplementary Fig. S3). This allows for future experiments at low passage numbers without significant genetic drift. Our transcriptomic analyses of a second generation of subclones from an isogenic monoclonal cell line (Fig. 5) demonstrates that clonal isolation from a monoclonal parental cell line results in very low variability of transcript levels even after multiple passages.

There are several limitations to this study. Firstly, we conducted experiments focusing on a single cell line. However, since heterogeneity has been frequently observed in different cell lines^9–11,47^, our findings are likely to apply to other cell lines as well. Secondly, investigated KO effects are restricted to a single gene (*Pkd1*). Yet, the underlying problem of WT heterogeneity is independent of the gene targeted via genome editing. Thus, similar findings are to be expected when targeting other genes.

In summary, this study identifies heterogeneity of WT cell lines as a significant cause for phenotypic variability of genome-edited cells. In addition, we demonstrate and validate an improved protocol for genome editing of stable cell lines.

## Methods

### Cell culture

mIMCD-3 wild-type cells were obtained from ATCC (Manassas, VA, USA, CRL-2123; Lot 5064816). Cells were cultured with DMEM:F12 (LONZA, cat.no. BE12-719F), fetal bovine serum was added to a final concentration of 10% and penicillin/streptomycin (Sigma, P0781) with a final concentration of 1%. Subculturing of cells was performed twice a week in a 1:10 ratio. Cells were maintained in a humidified incubator at 37°C and 10% CO_2_. Experiments were strictly performed with comparable and lowest possible passage numbers. In this manuscript, the term “wild-type” is used for mIMCD-3 cells that were not edited by CRISPR-Cas9. Of note, the original cell line obtained from ATCC was generated from an SV-40 transgenic mouse^37^.

### Protein isolation, SDS-PAGE, Western blot and ECL detection

Cells were harvested five days after epithelial confluency. Proteins were isolated and processed as described previously^48,49^. Chemiluminescence was detected by a 16-bit ChemoCam system (Intas). Images were acquired within the dynamic range of the charge-coupled device sensor. None of the analyzed bands was saturated. Antibodies used for protein detection: anti-YAP (Cell Signaling, 4912), anti-CaMKII delta (Abcam, ab181052), anti-pPERK (Cell Signaling, 3179), anti-pAMPK (Cell Signaling, 2535), anti-AMPK (Cell Signaling, 2532) and anti-ß-actin (Sigma A1978). Primary antibodies were diluted 1:1000 (anti-ß-actin 1:5000). Secondary antibodies (anti-rabbit, GE Healthcare NA934; anti-mouse, Dako P0447) were used 1:10,000.

### Cell sorting

Cells were single cell sorted with a BD FACS Aria III into 96-wells (Greiner Bio one, flat bottom, transparent). Cells were maintained in a humidified incubator at 37°C and 10% CO_2_, media was changed weekly. After 2.5 weeks, growth of clones was confirmed by visualization under an inverted microscope. Cells were expanded into 6-wells and finally 100 mm dishes.

### Cytotoxicity assay

Cisplatin (Merck, 232120) and methotrexate (Merck, 454126) were dissolved in PBS and DMSO, respectively. 4 × 10^4^ mIMCD-3 cells were seeded into 384-wells (Greiner bio-one, 781098) in 20 µl media. 5 µl of compounds were added at day 1 at indicated concentrations and incubated for 72 h. Negative control for cisplatin was conducted by incubating cells with media and PBS used for solving cisplatin at highest concentration (2%). Negative control for methotrexate was conducted by incubating cells with media and DMSO used for solving methotrexate at highest concentration (0.1% DMSO). Positive control was conducted by incubating cells for 72 h with digitonin (1000 µg/ml). At day 4, 25 µl of Promega’s CellTiter-Glo 2.0 solution were added per well to assess cell viability. Further steps were performed according to the manufacturer´s protocol. Luminescence was detected by a Tecan Spark reader.

### Generation of *Pkd1* knockout cells

Generation of polyclonal mIMCD-3 *Pkd1* KO cells was described earlier^17^. For generation of monoclonal KO the same protocol using the same Nickase-expressing plasmid pX335-U6-Chimeric-BB-CBh-hSpCas9n (Addgene, cat. no. 42335) and the same gRNAs (Supplementary Fig S5) was applied. However, a single cell sorting step was introduced prior to CRISPR/Cas9 application. *Pkd1* null alleles were confirmed by genomic sequencing, as well as complete absence of the respective mRNA and protein.

### Kidney tubule organoid assay

Growth-factor reduced Matrigel (Corning, 356231) and collagen type I rat tail (Ibidi, 50201) were mixed on ice in a 1: 1.25 ratio. 350 µl of this mixture were diluted with 630 µl DMEM:F12 media without fetal bovine serum. 20 µl HEPES (Sigma, H 0887) and 10 µl HGF (4 ng/µl, Merck, GF116) were added. mIMCD-3 cells were harvested via trypsinization (0.25% trypsin), counted and transferred to the gel mixture (150 cells/µl gel mixture). 80 µl/well of cell-gel-mixture were seeded into 96-wells (Greiner bio-one, flat bottom, transparent), plates were transferred to an incubator (37 °C). After 30 min, 220 µl of DMEM:F12 with fetal bovine serum and HGF (40 ng/ml) were carefully added on top of the cell-gel-mixture. After incubation for 72 h (37°C), media was carefully removed and replaced by 220 µl DMEM:F12 with fetal bovine serum without HGF. After an additional 96 h, cells were fixed for immunofluorescence.

### Immunofluorescence

mIMCD-3 cells grown in a Matrigel-collagen scaffold for 7 days were fixed with 4% PFA (200 µl/well, 30 min). After two washing steps (PBS 200 µl/well, 10 min) and blocking (5% horse serum, 0.1% Triton-X in PBS, 1 h), primary antibody or fluorescent dyes were added for 1 h. After three further washing steps (PBS 200 µl/well, 3 min), samples were incubated with the secondary antibody for 30 min. After washing an additional three times, samples were stored in PBS until imaging. Antibodies and fluorescent dyes used were anti-acetylated tubulin antibody (Sigma, T6793) Hoechst 33342 (Thermo Fisher Scientific, H1399, 1:1000), Alexa Fluor 488 phalloidin (Invitrogen, A12379), and anti-mouse Alexa Fluor 488 (Molecular Probes, A-11029).

### Organoid microscopy and image analysis

Kidney tubule organoids were visualized using a confocal microscope (Zeiss LSM-880). 300 µm z-stacks were imaged per well and fused into 2D images (Maximum intensity projection, Zeiss Zen blue). Structures per 2D image were categorized by morphology: Round structures were classified as spheroids, elongated structures > 30 µm were classified as tubules.

### RNA sequencing and data analysis

Total RNA of cells was extracted and sequenced at Novogene Co., Ltd. Sequencing of four biological replicates for each polyclonal and monoclonal cell lines was performed on an Illumina sequencer. Polyclonal cells were sequenced as 50 bp single-end reads and monoclonal cells as paired-end 150 bp reads. The raw sequencing data were quality controlled by Novogene and reads containing adapters and low quality base which is over 50% of the total bases were removed. The final fastq files were then aligned to mouse reference genome sequence GRCm38 using STAR 2.7.3a with default parameters^50^. The genome sequence and gene annotation files were downloaded from the Ensembl release 81. RSEM v1.3.1 was used to calculate gene expression values, which were represented as TPM (Transcripts Per Kilobase Million)^51^. Principal components analysis of the samples was performed using the R function prcomp on the log_2_(TPM) values of TPM. In order to perform differential gene expression analysis between polyclonal and monoclonal samples, which were sequenced at different time points, log_2_(TPM) values were z-score transformed. Welch t-test was used to compare gene expression between polyclonal and monoclonal samples as well as the monoclonal parental cell line with second-generation clones (two-sided comparison). Genes with Benjamini–Hochberg adjusted p-value < 0.01 and a z-score difference of < 1 or > 1 were defined as differentially expressed genes. Differentially expressed genes were tested for enrichment of Gene Ontology terms by the R package clusterProfiler v3.18.1 with default settings^52^.

### Multicolor FISH

Metaphases were prepared from mICMD-3 cells according to standard procedure^53,54^. The analysis was carried out using a 24XCyte Multicolor FISH probe (mFISH) (Metasystems, Altlussheim, Germany) and the analysis was performed according to the manufacturer´s instructions. The ISIS software (V. 5.10.129) was used for analysis (Metasystems, Altlussheim, Germany; https://metasystems-international.com/). Whenever possible, 5 metaphases were analyzed per sample.

### Statistics

All results are expressed as mean ± SEM. A p-value < 0.05 was considered significant unless otherwise specified. Statistical analysis was performed as described in the figure legends. Software used for statistics: Graph Pad Prism 8 for Windows and R v4.0.5 for Linux. Densitometry was performed with ImageJ 1.46R. For quantitative analysis, the intensity of the protein of interest band was normalized to the intensity of the corresponding actin band.

## Supplementary Information

Supplementary Information 1.

Supplementary Information 2.

Supplementary Information 3.

Supplementary Information 4.

## Data Availability

RNA-sequencing data generated and analyzed during the current study will be available in the gene expression omnibus database under the Accession Number: GSE196762. All other datasets generated and/or analyzed during the current study are available from the corresponding author on reasonable request.
