# Supplementary material for: Wildtype heterogeneity contributes to clonal variability in genome edited cells
Source: Sci Rep. 2022 Oct 28;12:18211. doi: 10.1038/s41598-022-22885-8 (PMC9616811; doi:10.1038/s41598-022-22885-8)
Supplement: Supplementary file 1 — Supplementary Information 1. [file 41598_2022_22885_MOESM1_ESM.pdf]

Supplementary Information File

Wildtype heterogeneity contributes to clonal variability in genome edited cells

Lukas Westermann[1], Yong Li[2], Burulca Göcmen[2], Matthias Niedermoser[1], Kilian Rhein[1], Johannes Jahn[1], Isabel Cascante[1], Felix Schöler[1], Niklas Moser[1], , Björn Neubauer[1], Alexis Hofherr[1], Yvonne Lisa Behrens[4], Gudrun Göhring[4], Anna Köttgen[2,3], Michael Köttgen[1,3] * & Tilman Busch[1]

Affiliation:

1. Renal Division, Department of Medicine, Medical Center, Faculty of Medicine, University of Freiburg, Freiburg, Germany.
2. Institute of Genetic Epidemiology, Faculty of Medicine and Medical Center - University of Freiburg, Freiburg, Germany.
3. CIBSS - Centre for Integrative Biological Signalling Studies, Freiburg, Germany.
4. Department of Human Genetics, Hannover Medical School, Hannover, Germany.

Supplementary Figure S1

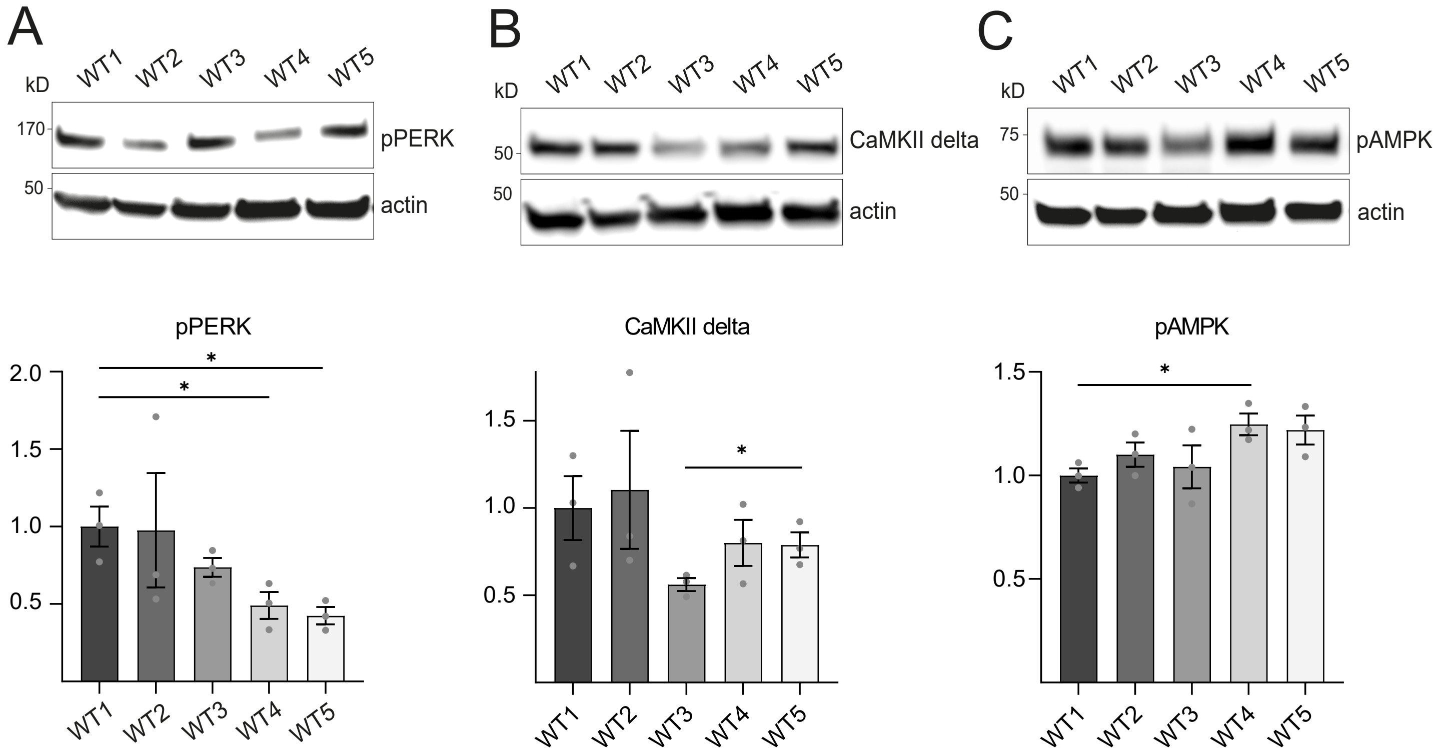

Supplementary Fig. S1: Monoclonal mIMCD-3 wild-type cells vary in protein levels of three protein kinases.

(**A**) Protein abundance of pPERK varies in five different monoclonal mIMCD-3 WT cells after Western blotting. Densitometry shows a significant decrease in protein abundance of pPERK in WT4 and WT5 compared to WT1. WT5 also shows a significant decrease in protein abundance compared to WT3 (**B**) Protein abundance of CaMKII delta varies in five different monoclonal mIMCD-3 WT cells. Densitometry shows a significant decrease in protein abundance of CaMKII delta in WT3 compared to WT5. (**C**) Protein abundance of pAMPK varies in five different monoclonal mIMCD-3 WT cells. Densitometry shows a significant increase in protein abundance of pAMPK in WT4 compared to WT1. Three independent experiments were included in each analysis of protein abundance. Error bars represent SEM. Statistical significance was evaluated using unpaired t-test. * indicates $p < 0.05$. Unproccessed blots (**A-C**) are presented in Supplementary Fig. S12, S13, and S14.

Supplementary Figure S2

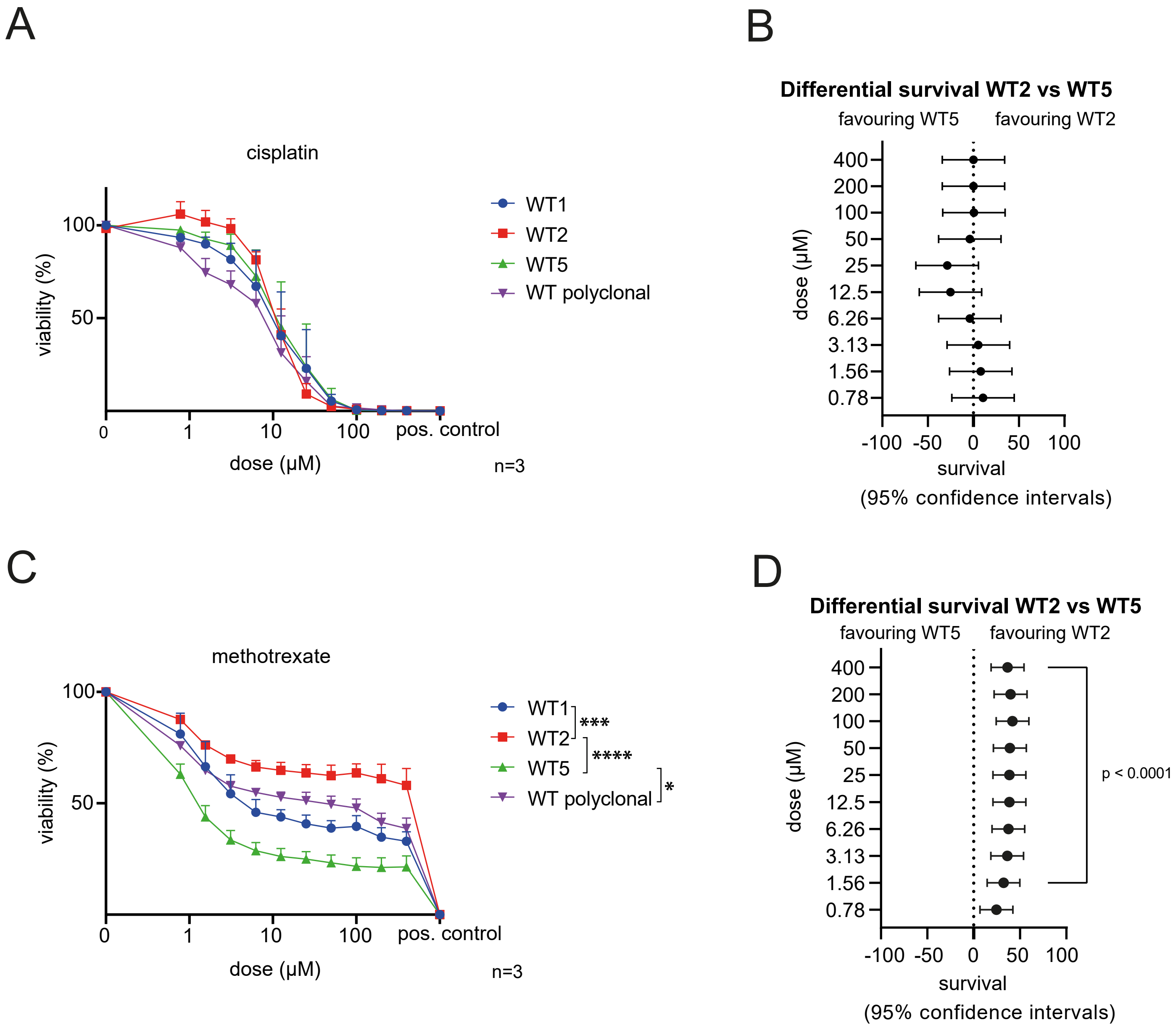

Supplementary Fig. S2: Monoclonal mIMCD-3 wild-type cells differ in viability after treatment with cisplatin and methotrexate.
(**A**) Dose-response curves for mono- and polyclonal mIMCD-3 WT cell lines after incubation with cisplatin for 72 hours at indicated concentrations. Viability was indirectly measured by detecting luminescence. 100% viability was defined as negative (solvent) control. 0% viability is defined as positive control signal. Each data point represents mean luminescence signal normalized to corresponding negative control from three independent experiments. Error bars represent SEM. (**B**) Differential viability between WT2 and WT5 is illustrated by comparing 95% confidence intervals (Sidak correction) for indicated concentrations. Cell lines tend to show different, but non-significantly altered viability at 12.5 and 25 µM cisplatin. (**C**) Dose-response curves for mono- and polyclonal mIMCD-3 WT cell lines after incubation with methotrexate for 72 hours at indicated concentrations. Cell lines show significant differences in viability in response to methotrexate treatment. Significant differences are indicated (* = $p < 0.05$; *** = $p < 0.001$; **** = $p < 0.0001$). (**D**) Differential viability between WT2 and WT5 is illustrated by comparing 95% confidence intervals. Cell lines differ significantly in viability after treatment with methotrexate at all indicated concentrations.

Supplementary Figure S3

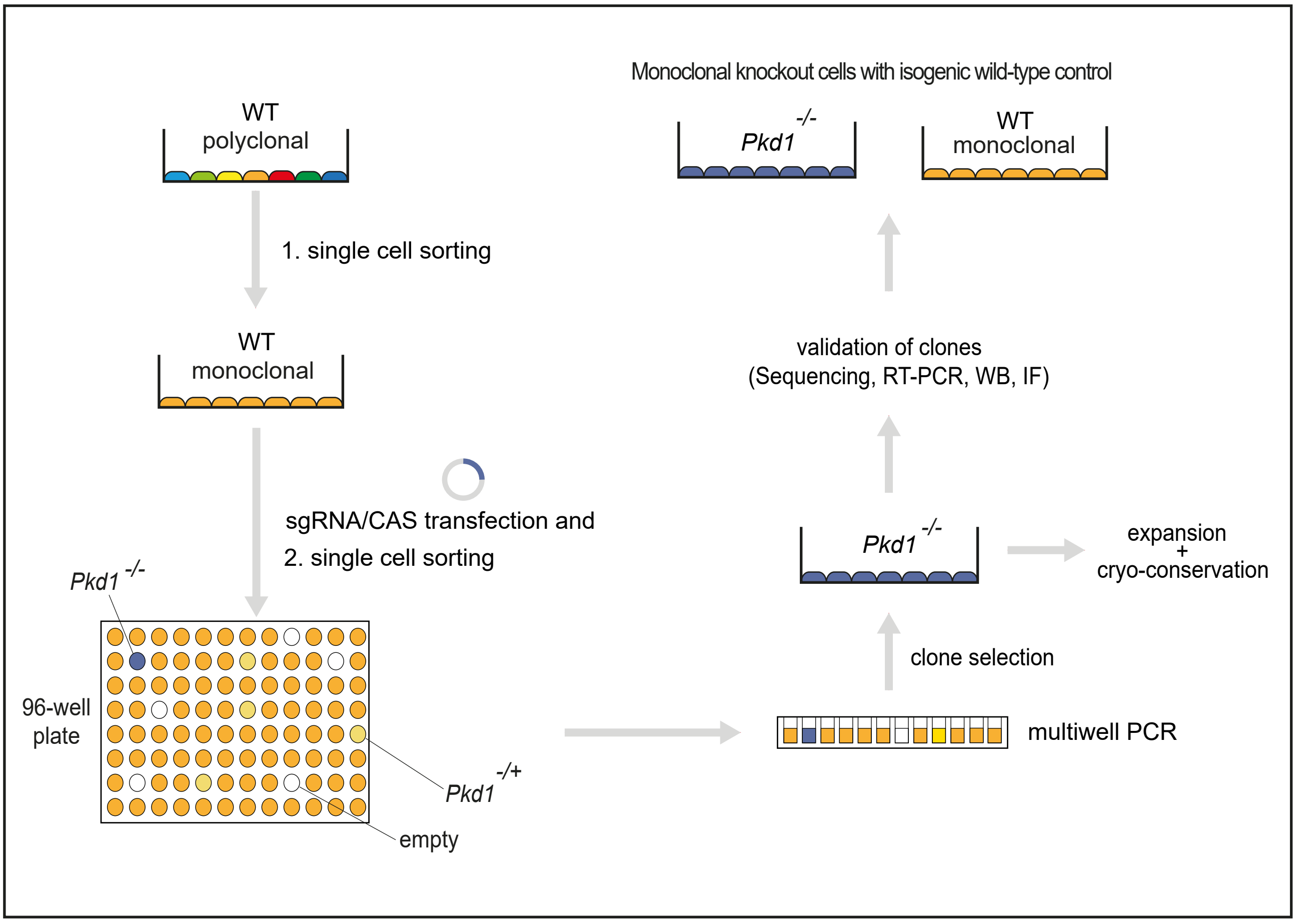

Supplementary Fig S3: Improved protocol for knockout cell line generation.
Our data shows that mIMCD-3 cells are polyclonal in nature. Hence, an additional cell sorting step is implemented in an improved CRISPR protocol. First, polyclonal WT cells are single cell sorted. Resulting monoclonal WT cells are transfected with Cas9 and gRNAs. A second step of single cell sorting is performed. Genomic identity can be assessed by multiwell PCR. Possible hits are validated. Potential candidate clones are cryo-conserved as soon as possible. In case of successful validation, these clones are expanded and cryo-conserved for long-term storage. The additional sorting step tas shown in this illustration results in the generation of a monoclonal KO cell line with an isogenic WT control.

Supplementary Figure S4

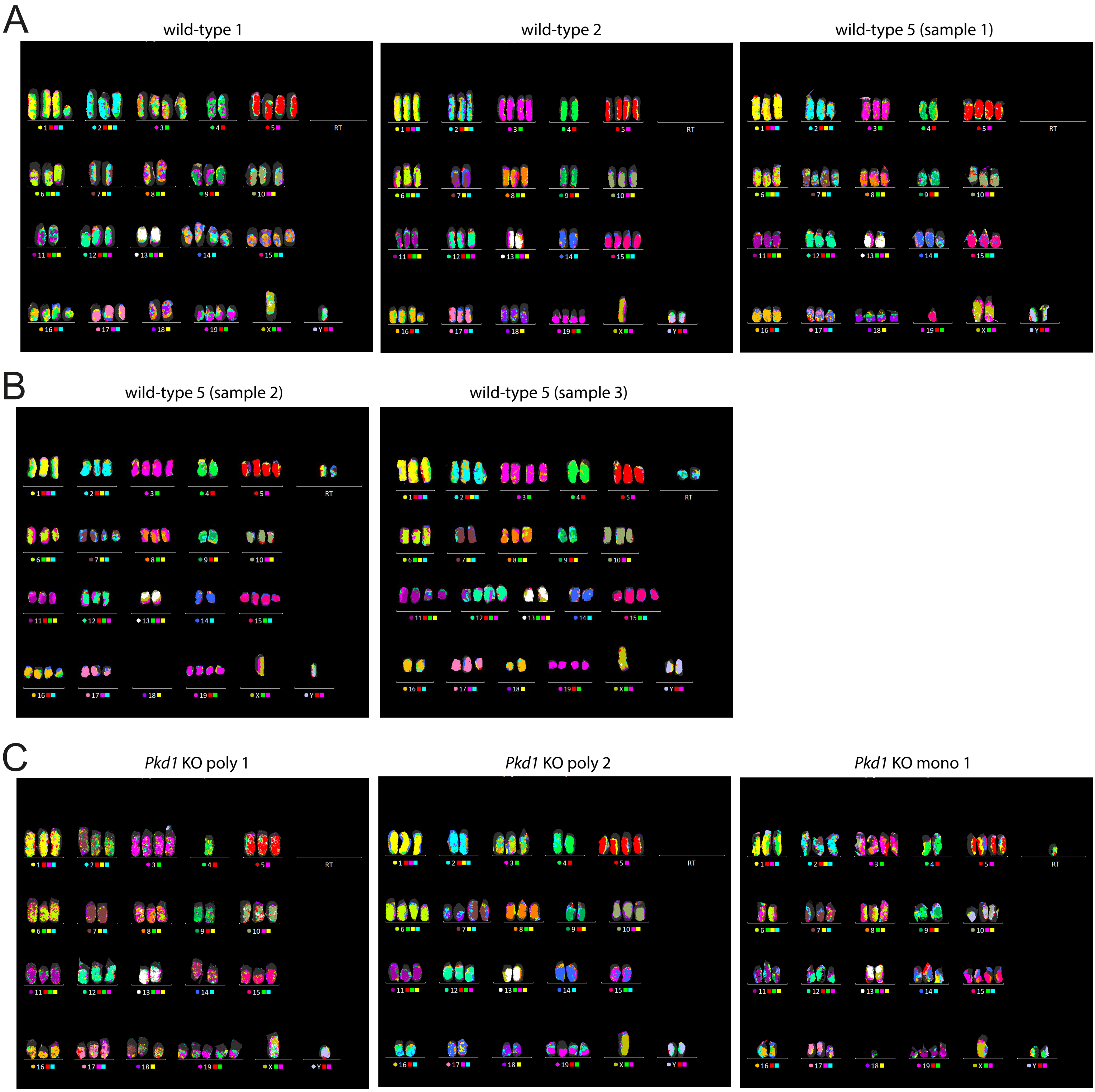

Supplementary Fig. S4: Multicolor fluorescence in situ hybridisation analysis of monoclonal and polyclonal mIMCD-3 cell lines demonstrates similar karyograms.
Illustrated are karyograms for (**A**) monoclonal wild-type cell lines, (**B**) technical replicates of monoclonal wild-type cell line 5 and (**C**) for two polyclonal *Pkd1* KO and one monoclonal *Pkd1* KO cell line. All investigated karyograms show polyploidy without evidence for structural aberrations or chromosomal instability.

Supplementary Figure S5

A

Cell line	Deleted sequence (GRCm39)
mIMCD-3 *Pkd1* KO polyclonal #1	17: 24769079 - 24813878 /<br>17: 24769028 – 24813938
mIMCD-3 *Pkd1* polyclonal #2	17: 24769060 – 24813883<br>17: 24769075 - 24813924
mIMCD-3 *Pkd1* KO monoclonal #1	17: 24769023 - 24813907
mIMCD-3 *Pkd1* KO monoclonal #2	17: 24769063 – 24813924

B

Cell line	gRNA binding site
gRNA *Pkd1* Exon 1 #1	TCTGGGGCAGGCCGCACCTC
gRNA *Pkd1* Exon 1 #2	GTCGCACCGCAGACGGGCCA
gRNA *Pkd1* Exon 45 #1	AGAGCTCGCCACGCAAGGCG
gRNA *Pkd1* Exon 45 #2	ACCCCAGGACTATGAGATGG

Supplementary Fig. S5: Overview of genomic data for generation of monoclonal and polyclonal *Pkd1* KO clones.
(**A**) Genomic data showing deleted sequences for indicated *Pkd1* KO cell lines. (**B**) gRNA binding sites used for generation of mIMCD-3 *Pkd1* KO cell lines.

Supplementary Figure S6

Figure 1A, YAP

YAP (approx. 70 kDa)

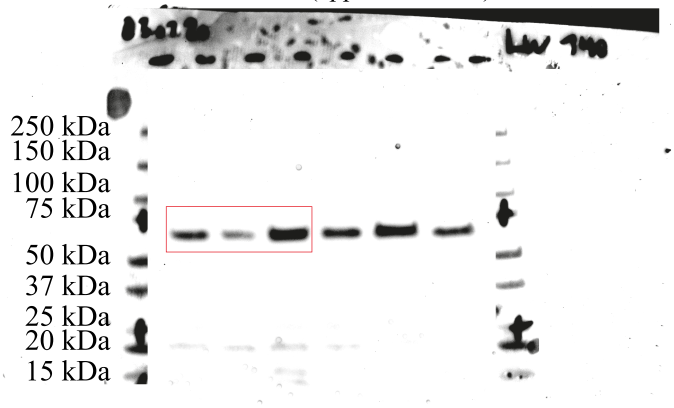

Figure 1A, actin

actin (approx. 45 kDa)

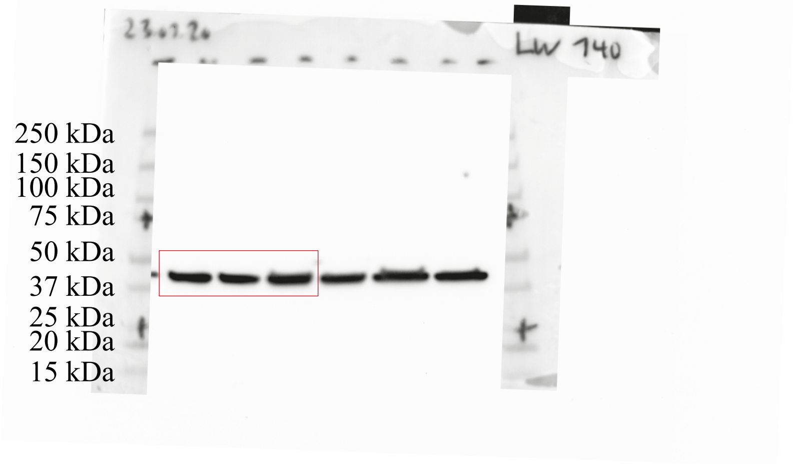

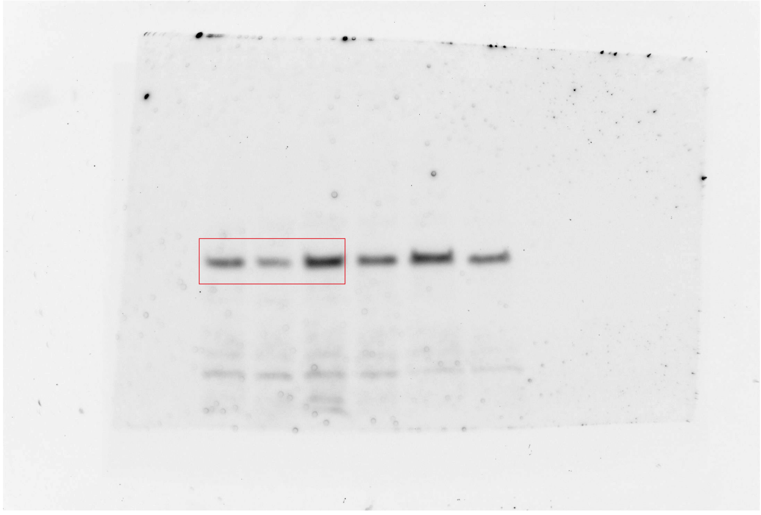

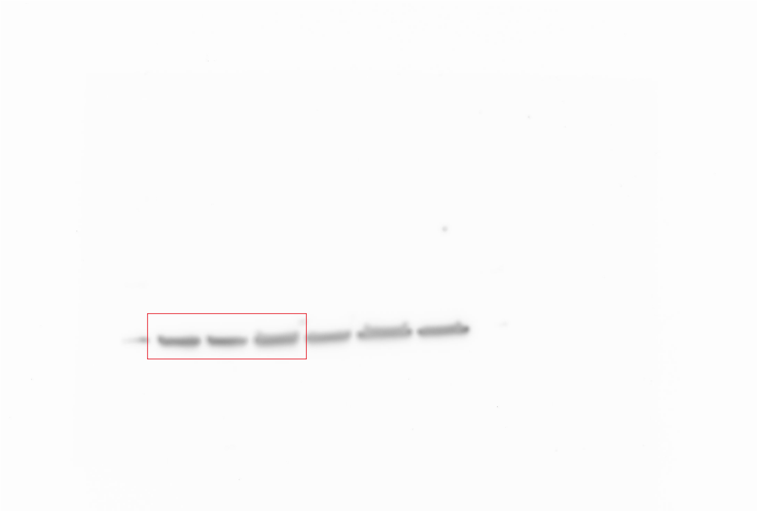

Supplementary Fig S6: Unprocessed blots of Figure 1A

Supplementary Figure S7

Figure 1D, YAP

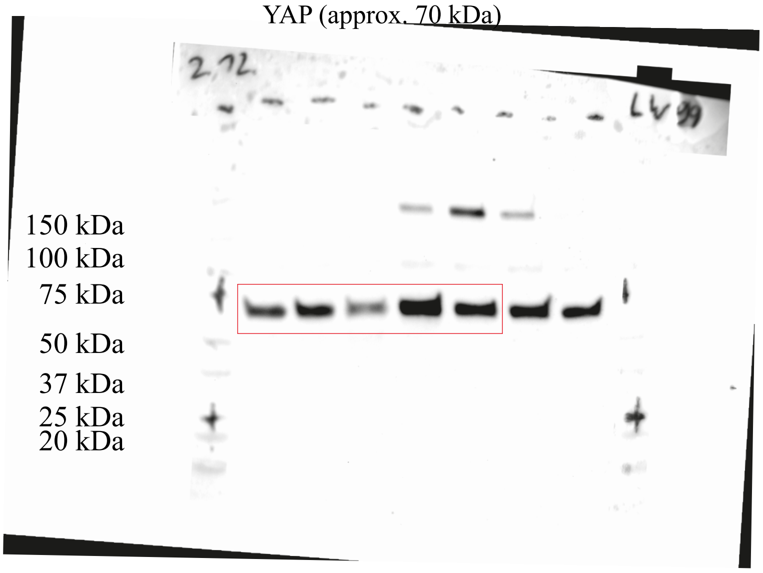

Figure 1D, actin

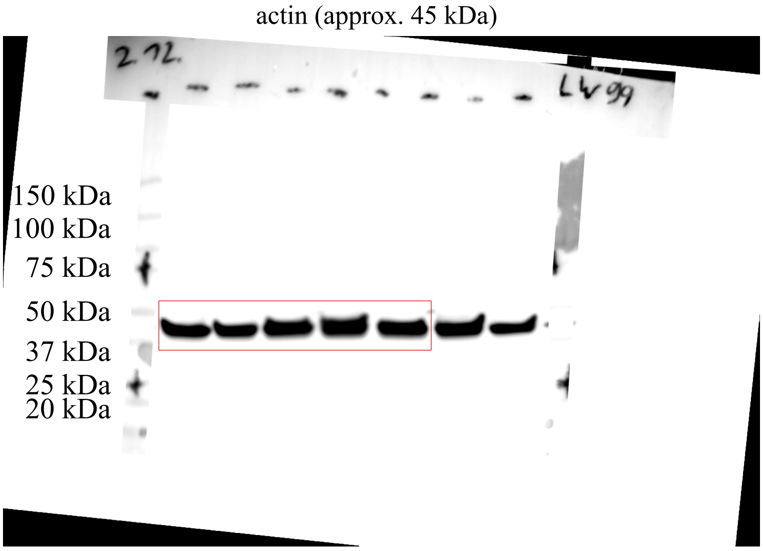

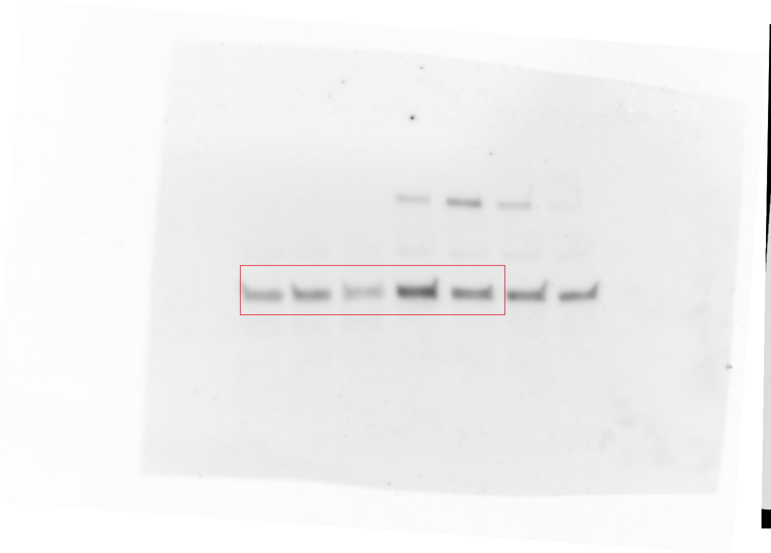

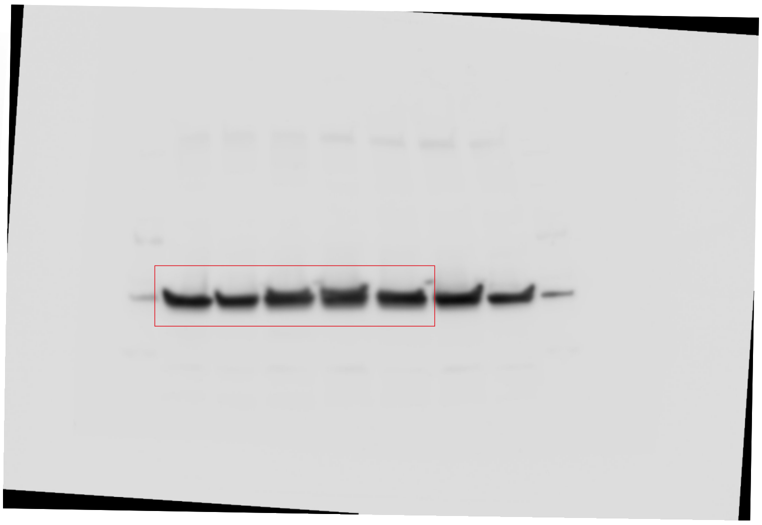

Supplementary Fig S7: Unprocessed blots of Figure 1D

Supplementary Figure S8

Figure 3A (left), AMPK

AMPK (approx. 62 kDa)

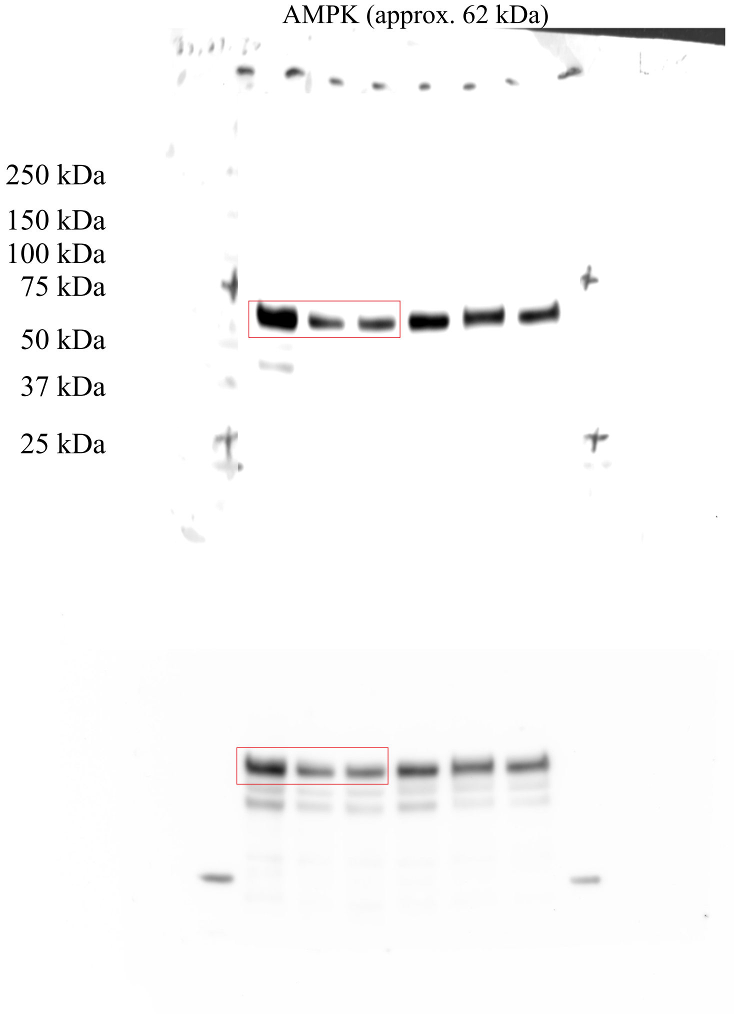

Figure 3A (left), actin

actin (approx. 45 kDa)

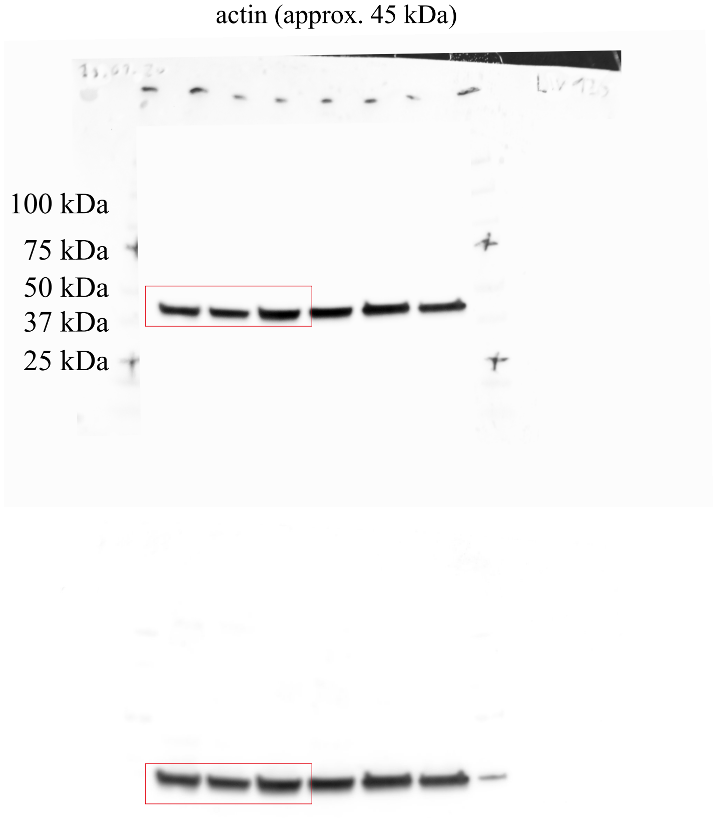

Figure 3A (right), AMPK

AMPK (approx. 62 kDa)

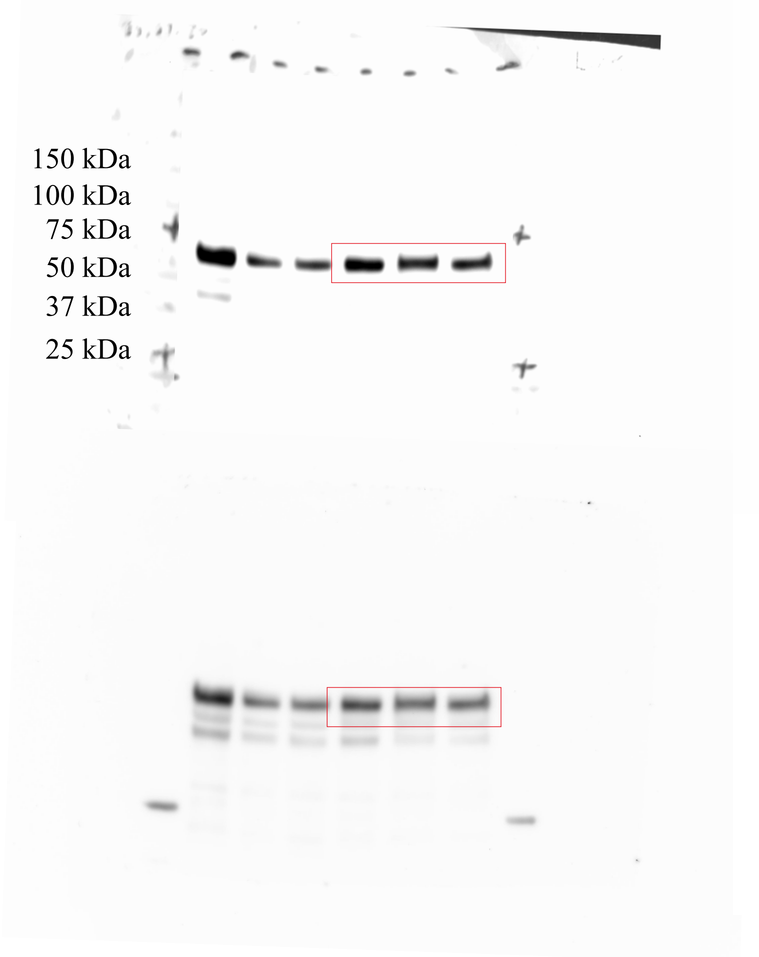

Figure 3A (right), actin

actin (approx. 45 kDa)

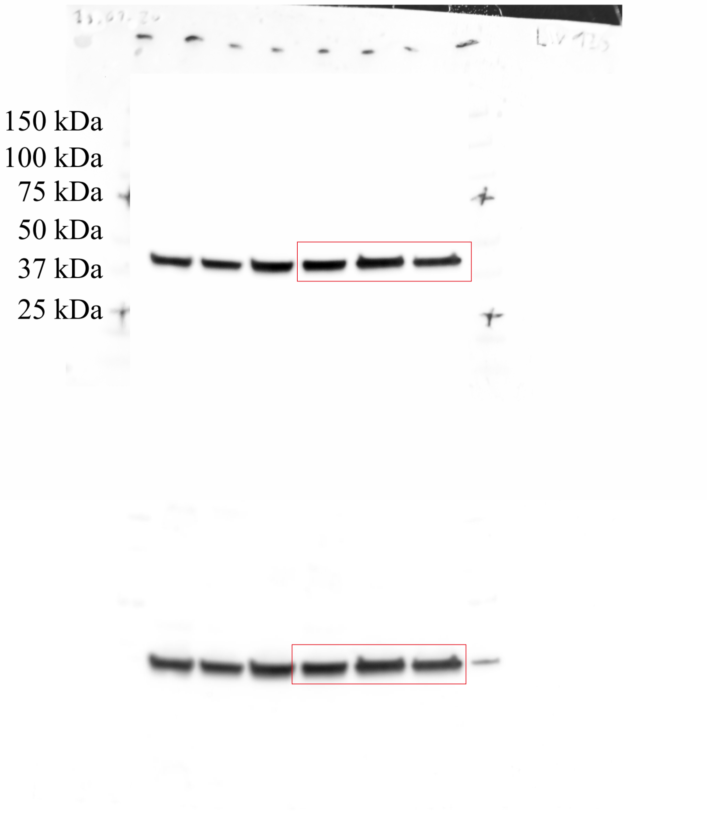

Supplementary Fig S8: Unprocessed blots of Figure 3A

Supplementary Figure S9

Figure 3B (left), pAMPK

pAMPK (approx. 62 kDa)

150 kDa
75 kDa
25 kDa

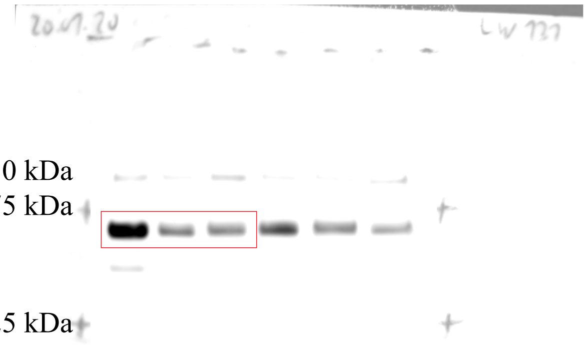

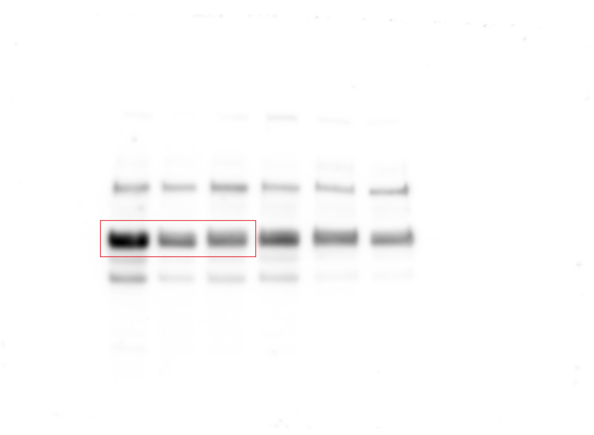

Figure 3B (left), actin

actin (approx. 45 kDa)

75 kDa
25 kDa

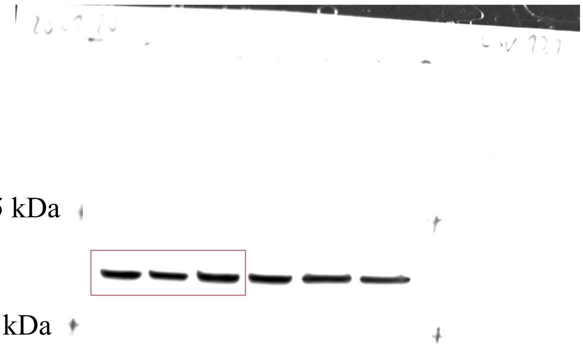

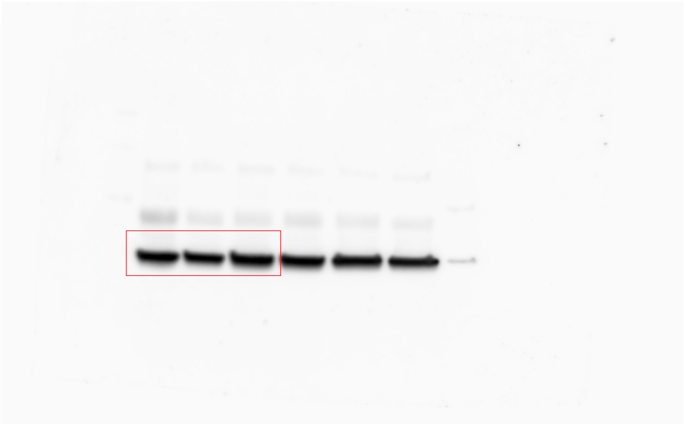

Figure 3B (right), pAMPK

pAMPK (approx. 62 kDa)

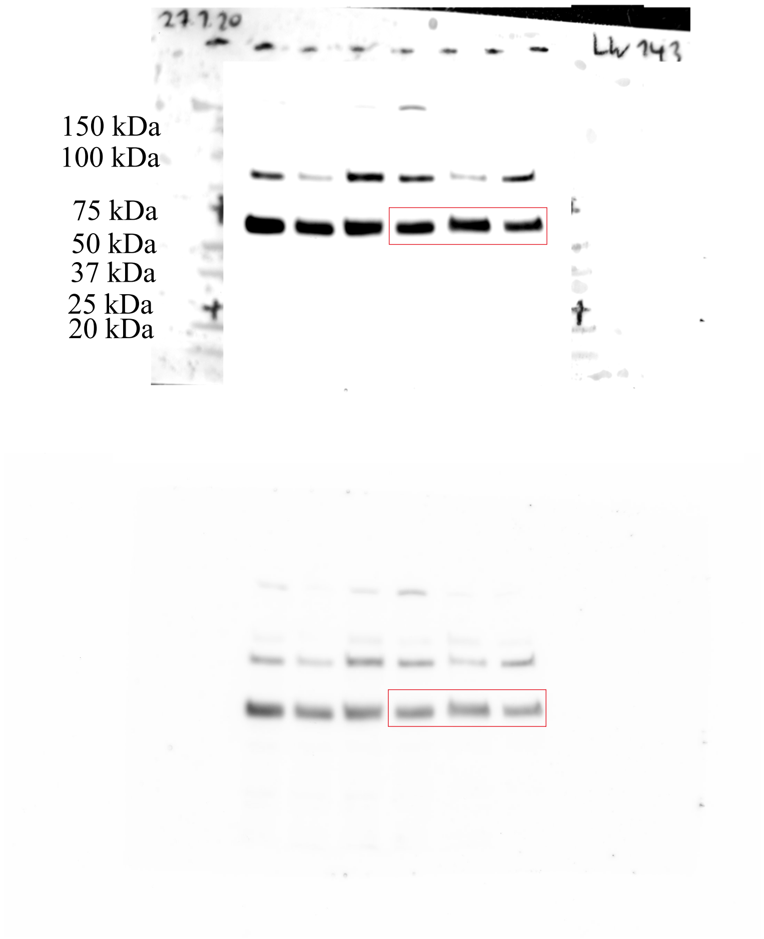

Figure 3B (right), actin

actin (approx. 45 kDa)

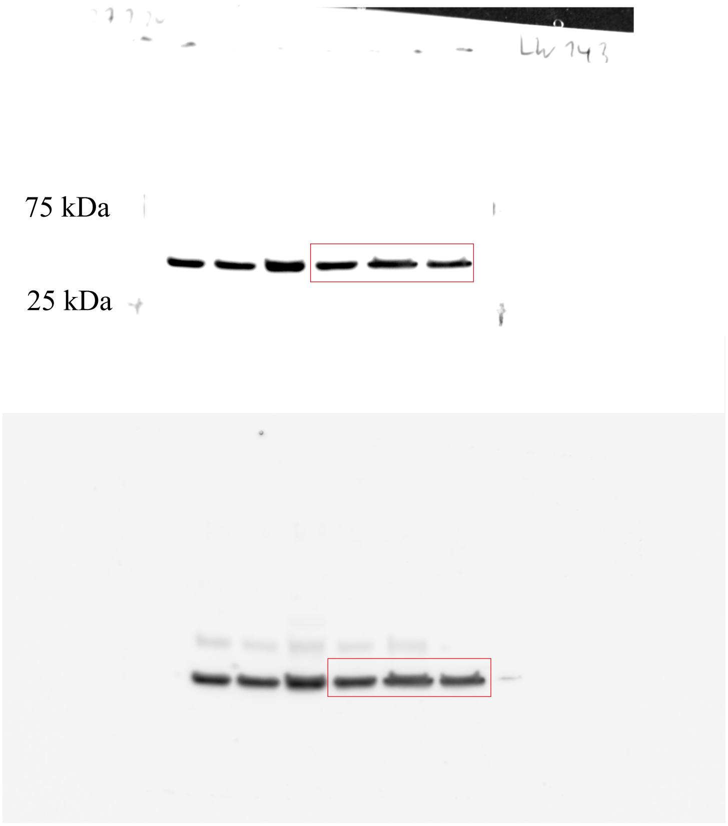

Supplementary Fig S9: Unprocessed blots of Figure 3B

Supplementary Figure S10

Figure 3C (left), CaMKII

CaMKII (approx. 50 kDa)

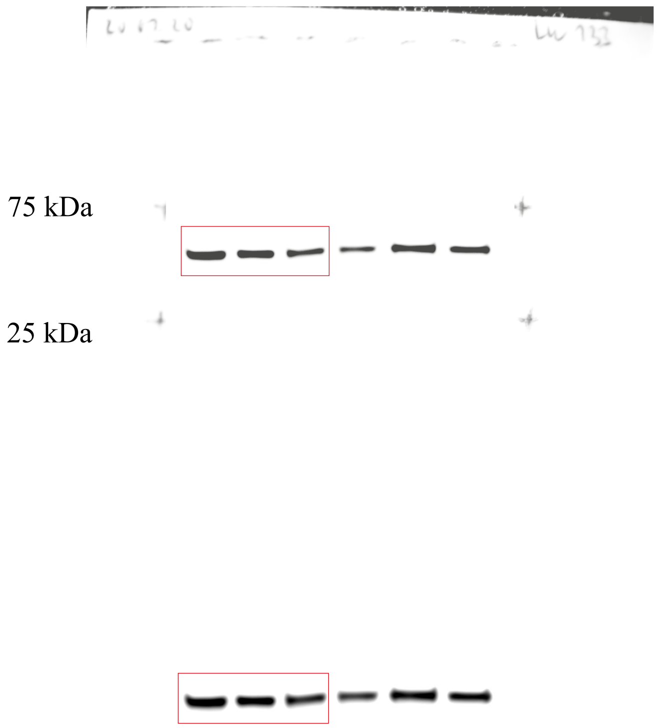

Figure 3C (left), actin

actin (approx. 45 kDa)

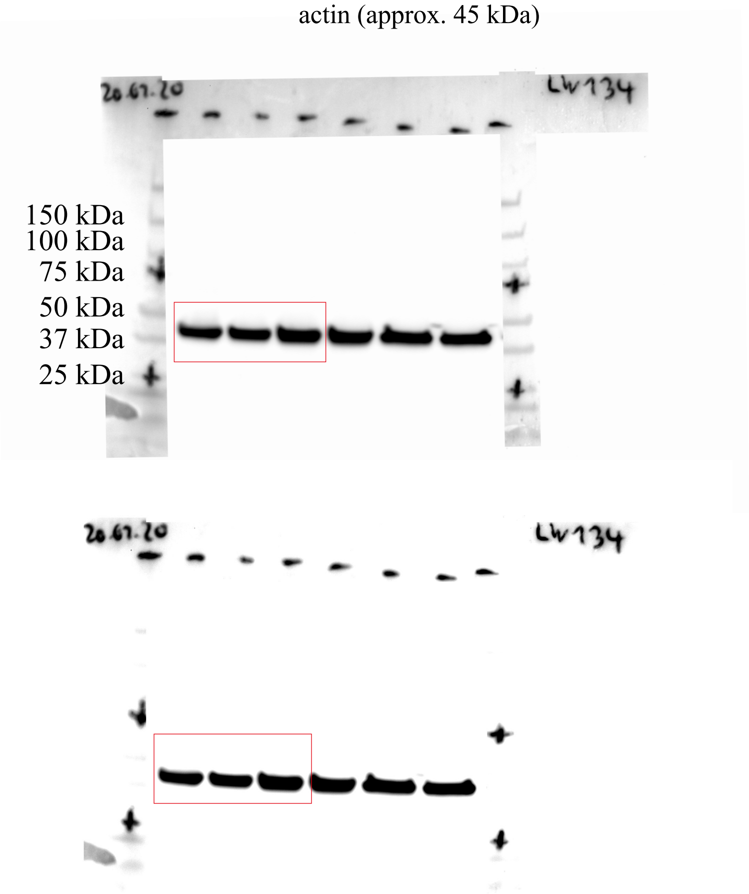

Figure 3C (right), CaMKII

CaMKII (approx. 50 kDa)

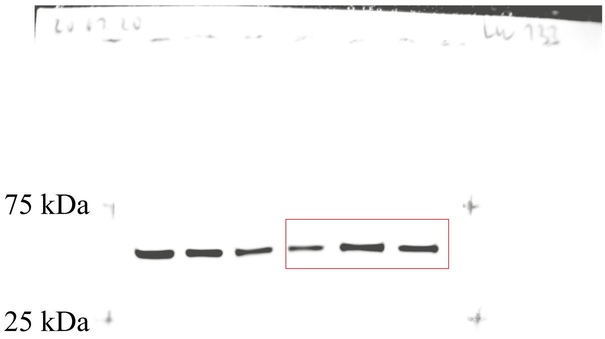

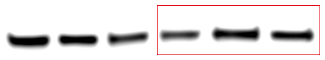

Figure 3C (right), actin

actin (approx. 45 kDa)

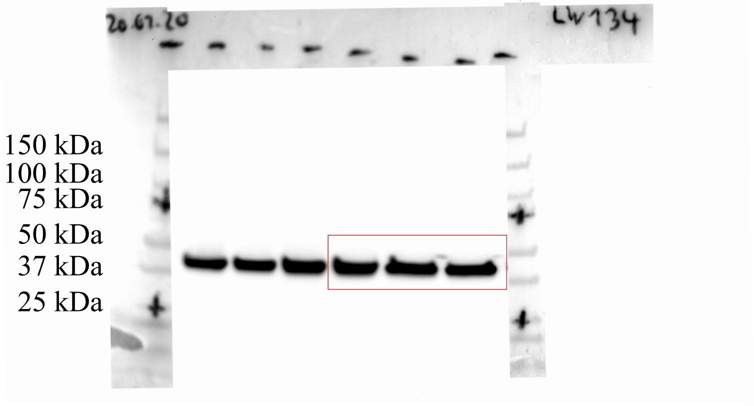

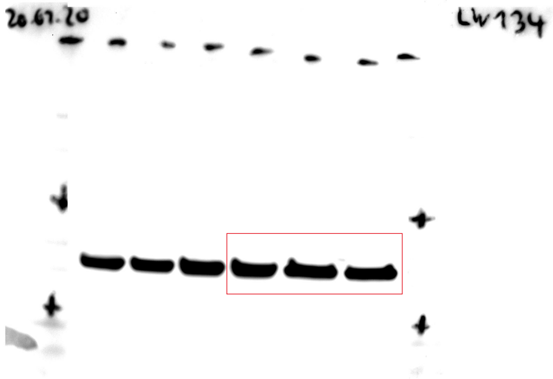

Supplementary Fig S10: Unprocessed blots of Figure 3C

Supplementary Figure S11

Figure 3D, YAP

YAP (approx. 70 kDa)

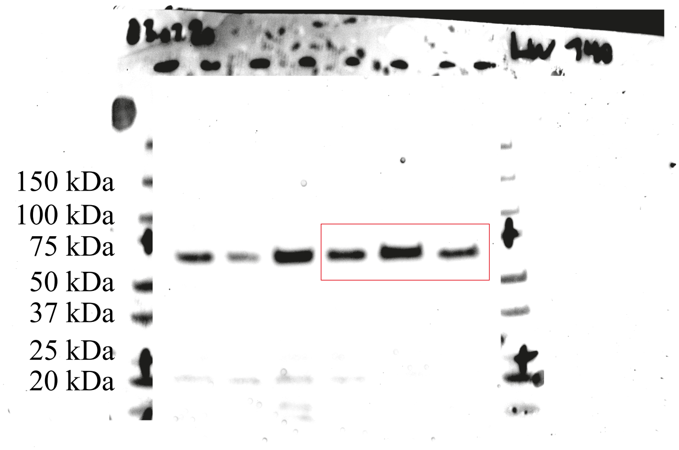

Figure 3D, actin

actin (approx. 45 kDa)

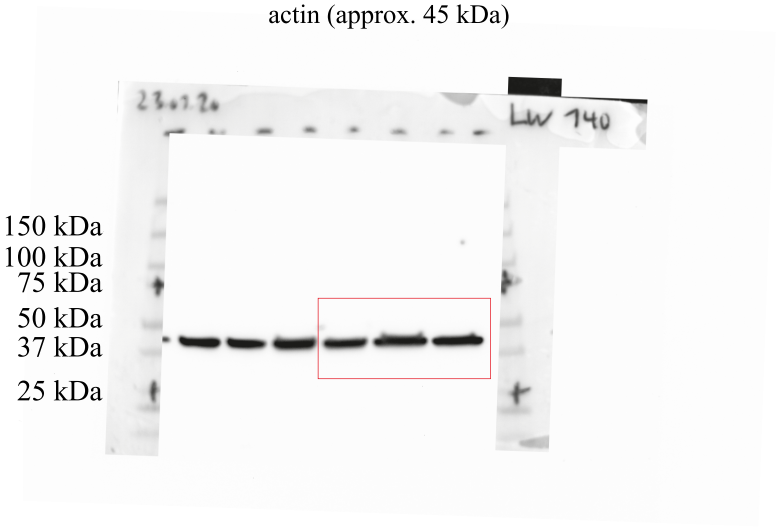

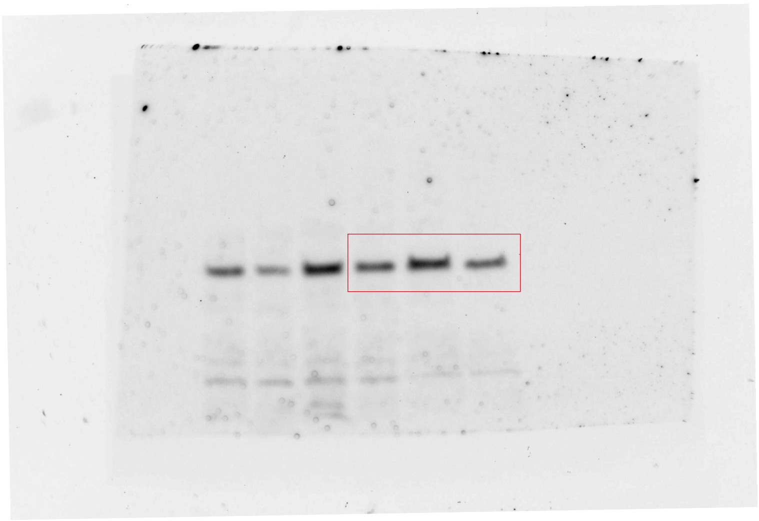

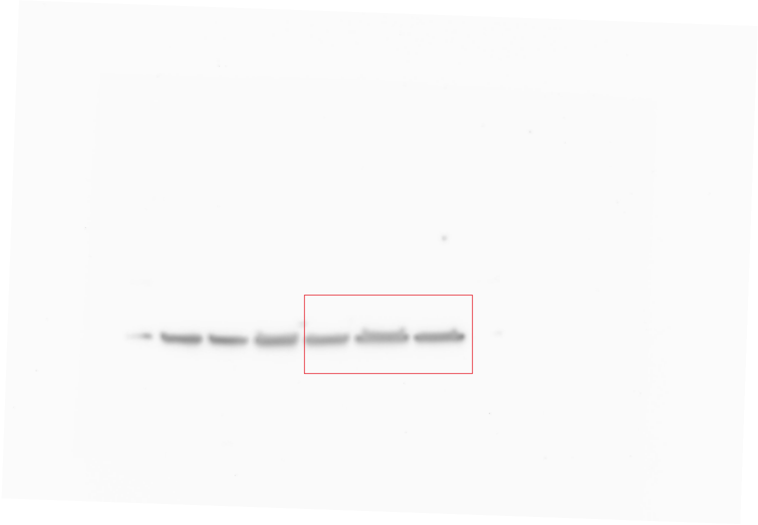

Supplementary Fig S11 : Unprocessed blots of Figure 3D

Supplementary Figure S12

Supplementary Figure 1A, pPERK

pPERK (approx. 170 kDa)

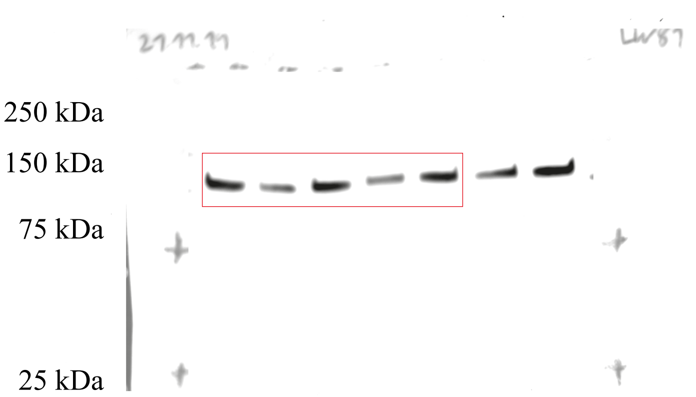

Supplementary Figure 1A, actin

actin (approx. 45 kDa)

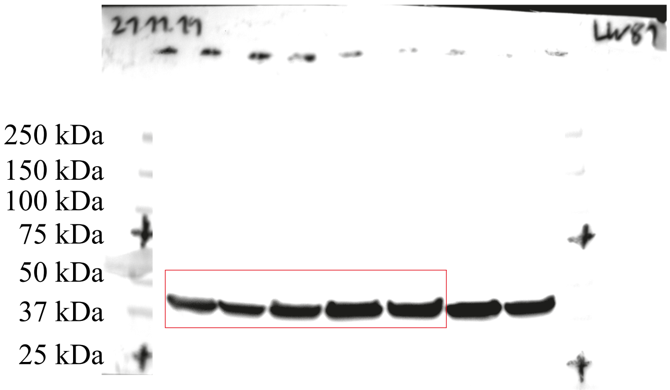

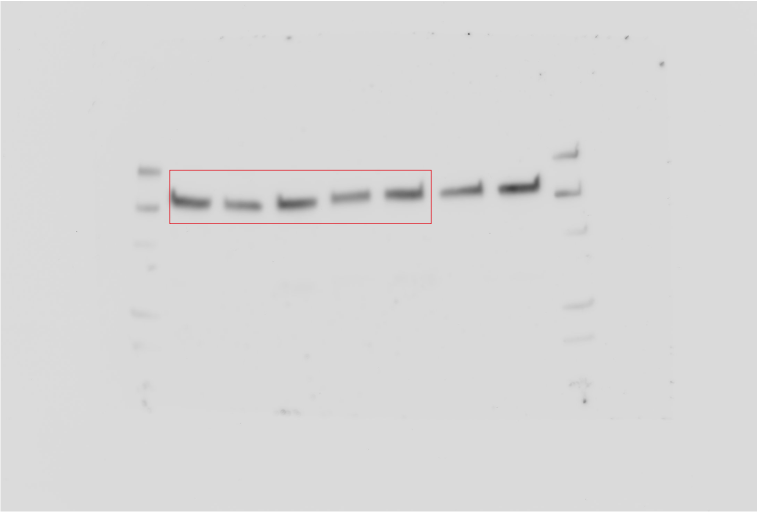

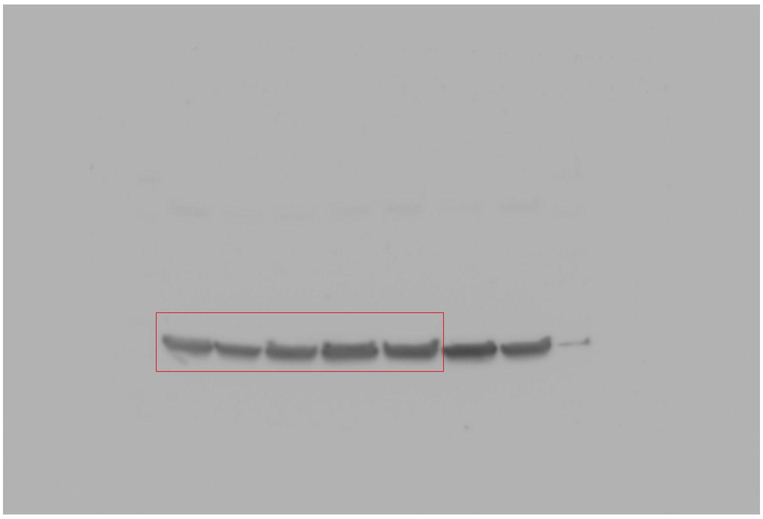

Supplementary Fig S12 : Unprocessed blots of Supplementary Figure S1 (A)

Supplementary Figure S13

Supplementary Figure 1B, CaMKII

CaMKII (approx. 50 kDa)

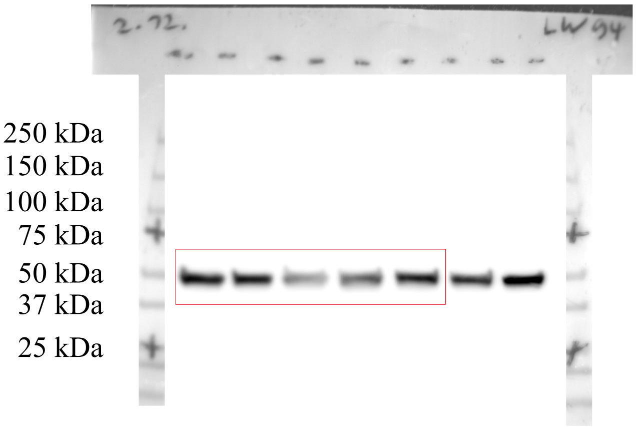

Supplementary Figure 1B, actin

actin (approx. 45 kDa)

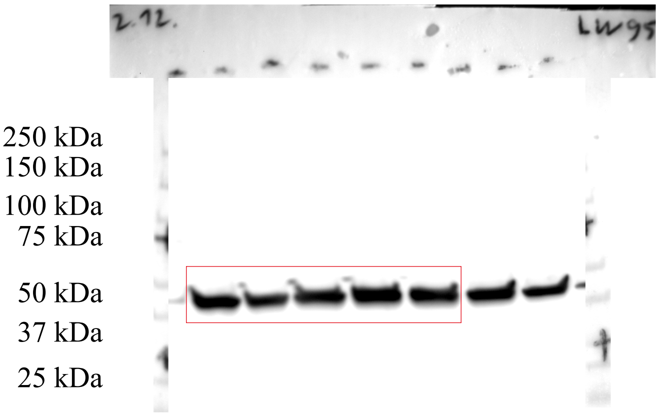

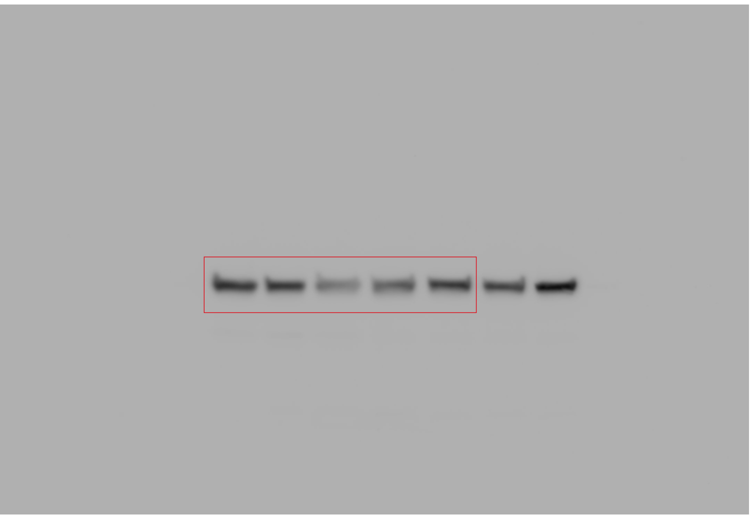

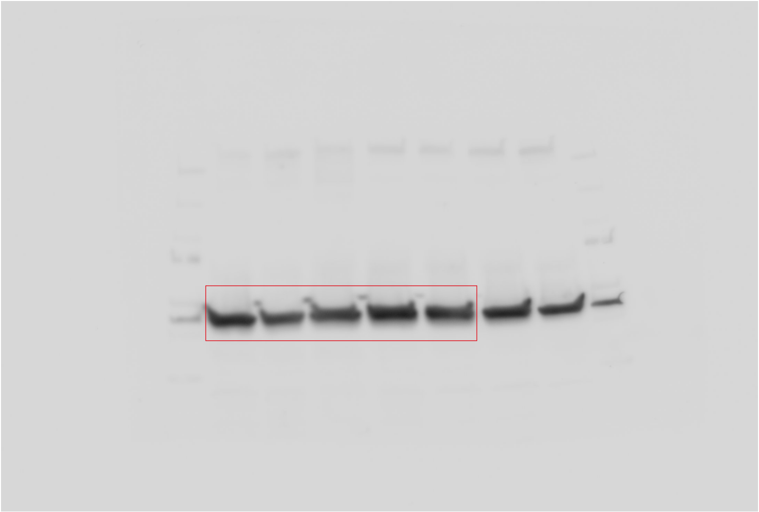

Supplementary Fig S13 : Unprocessed blots of Supplementary Figure S1 (B)

Supplementary Figure S14

Supplementary Figure 1C

pAMPK (approx. 62 kDa)

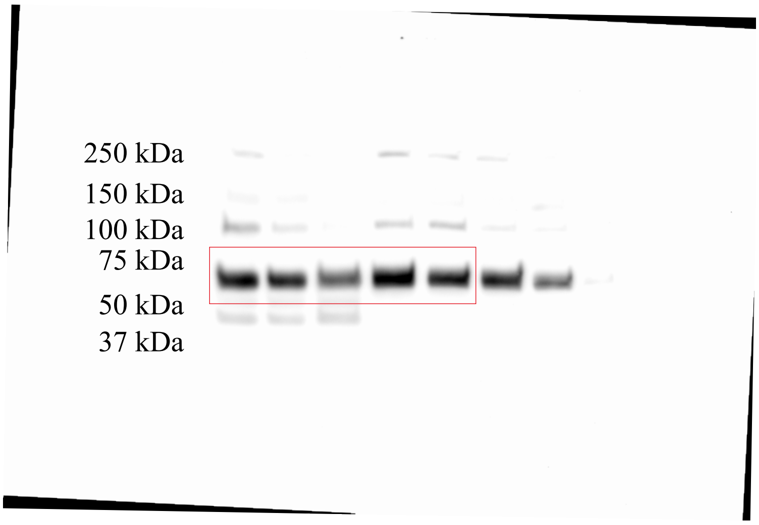

Supplementary Figure 1C, actin

actin (approx. 45 kDa)

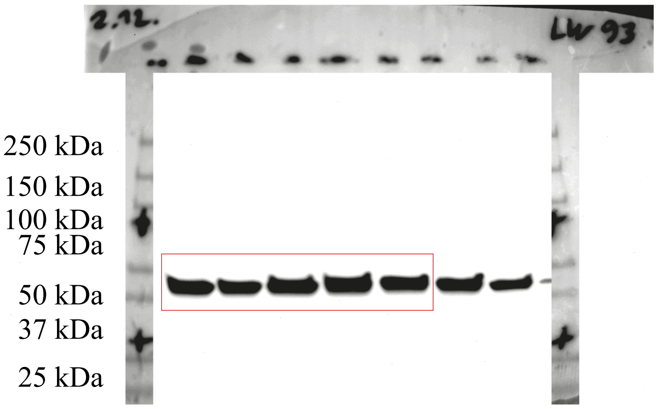

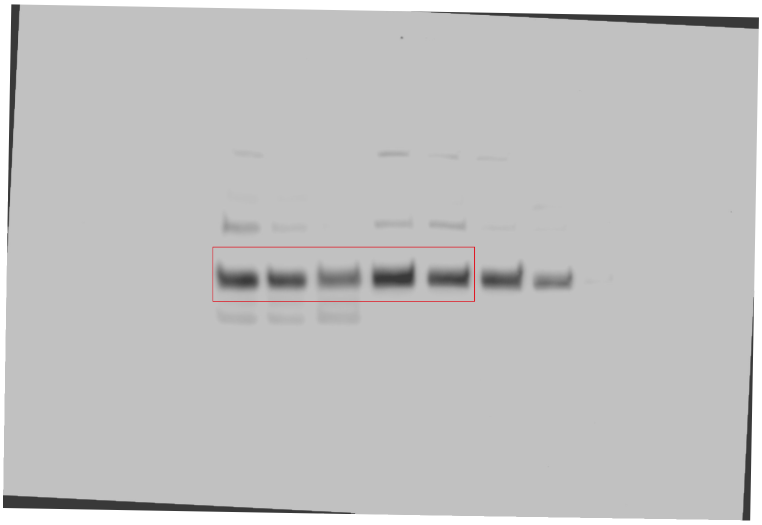

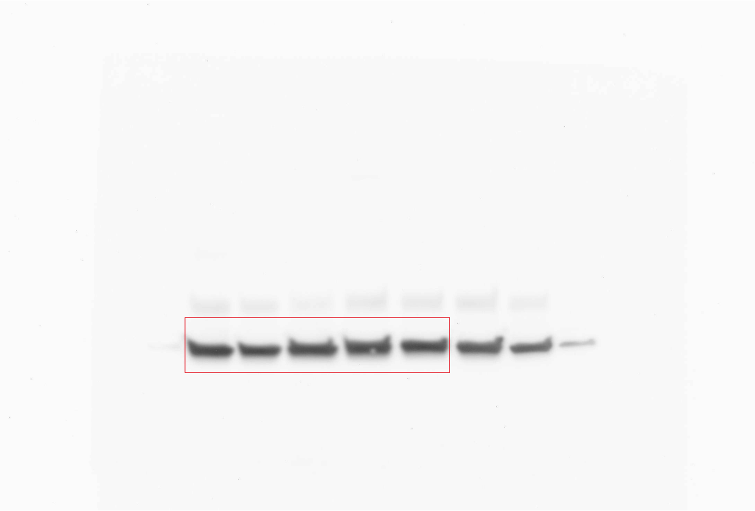

Supplementary Fig S14 : Unprocessed blots of Supplementary Fig S1 (C)

Supplementary Figure S15

n2

Figure 1A, YAP

YAP (approx. 70 kDa)

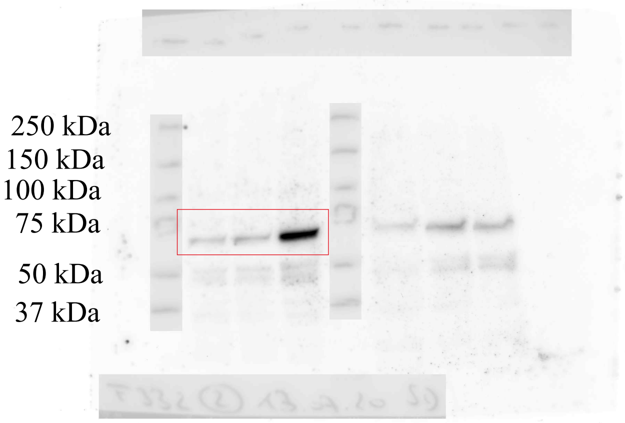

Figure 1A, actin

actin (approx. 45 kDa)

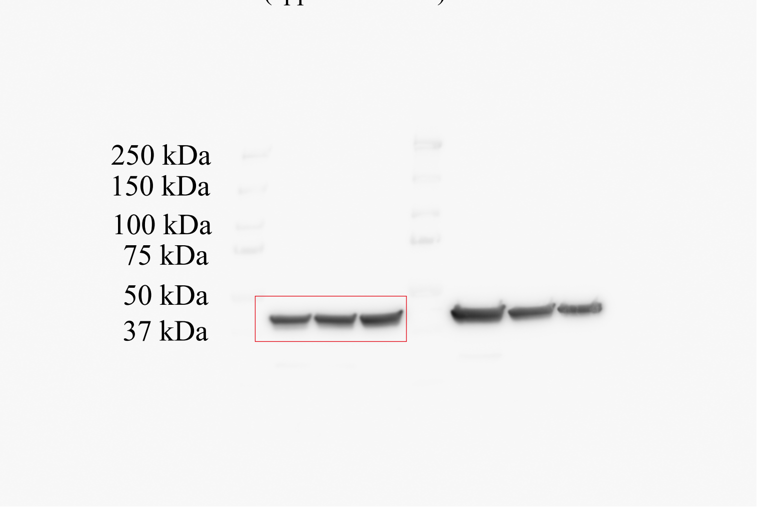

n3

Figure 1A, YAP

YAP (approx. 70 kDa)

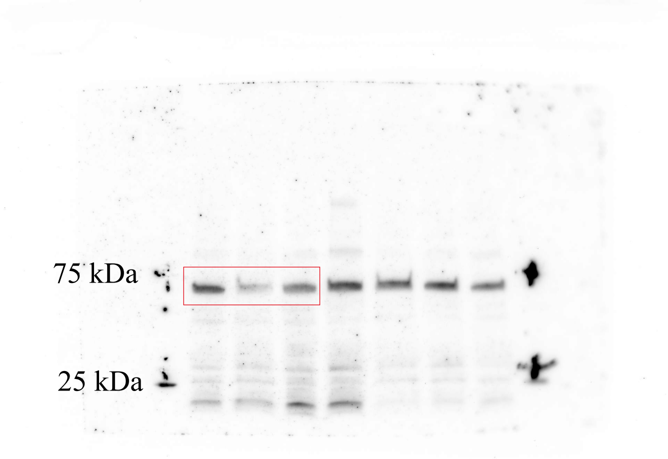

Figure 1A, actin

actin (approx. 45 kDa)

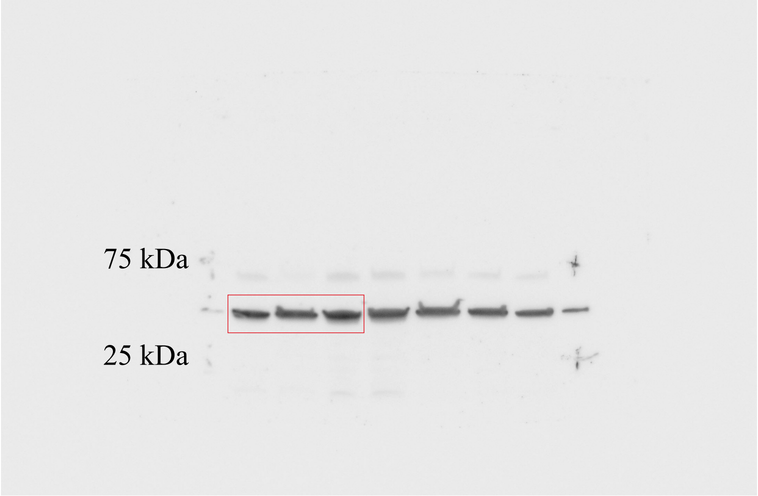

Supplementary Fig S15 : Unprocessed replicate blots of Figure 1A

Supplementary Figure S16

n2

Figure 1D, YAP

YAP (approx. 70 kDa)

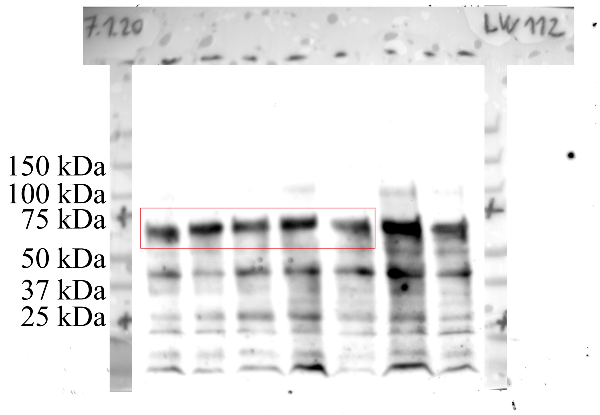

Figure 1D, actin

actin (approx. 45 kDa)

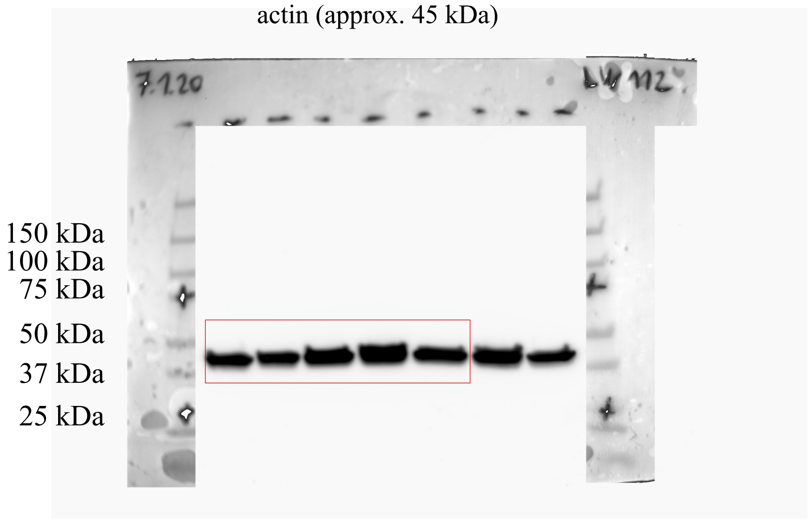

n3

Figure 1D, YAP

YAP (approx. 70 kDa)

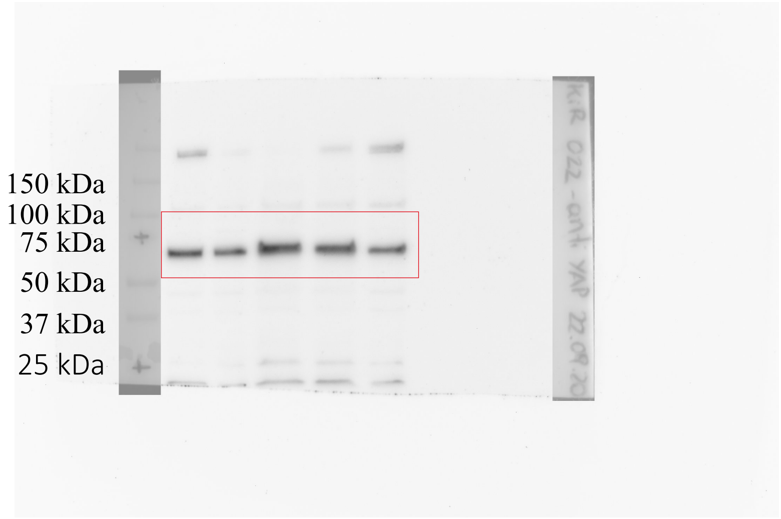

Figure 1D, actin

actin (approx. 45 kDa)

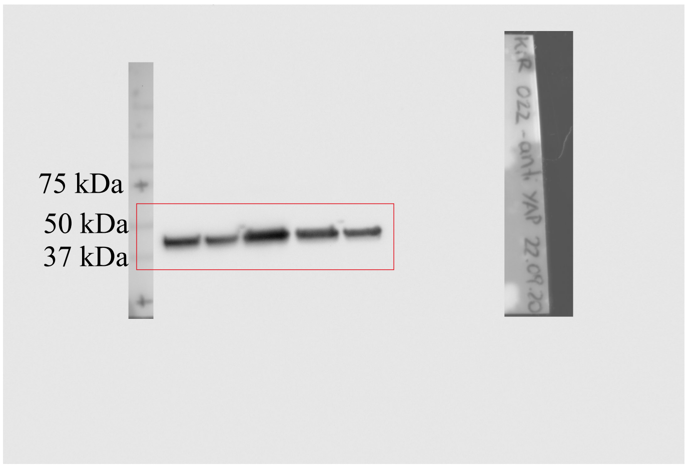

Supplementary Fig S16 : Unprocessed replicate blots of Figure 1D

Supplementary Figure S17

n2

Figure 3A (left), AMPK

AMPK (approx. 62 kDa)

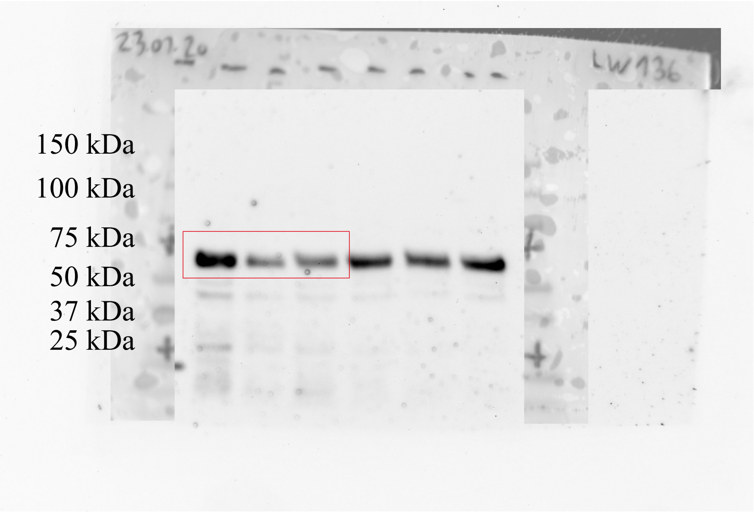

Figure 3A (left), actin

actin (approx. 45 kDa)

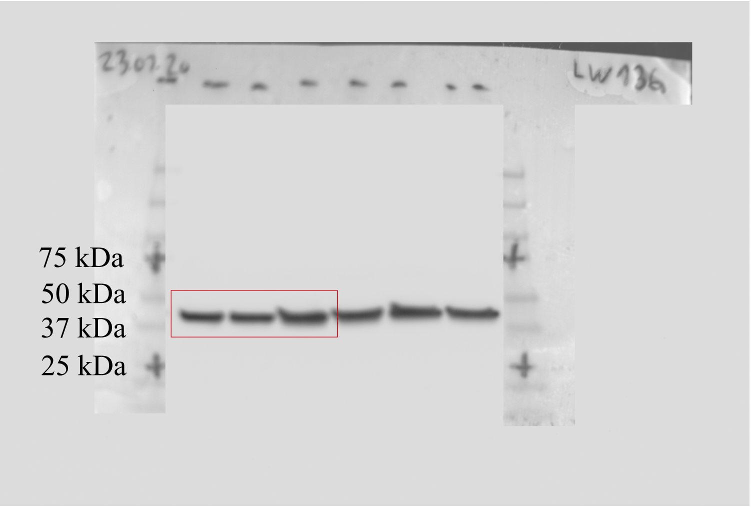

n3

Figure 3A (left), AMPK

AMPK (approx. 62 kDa)

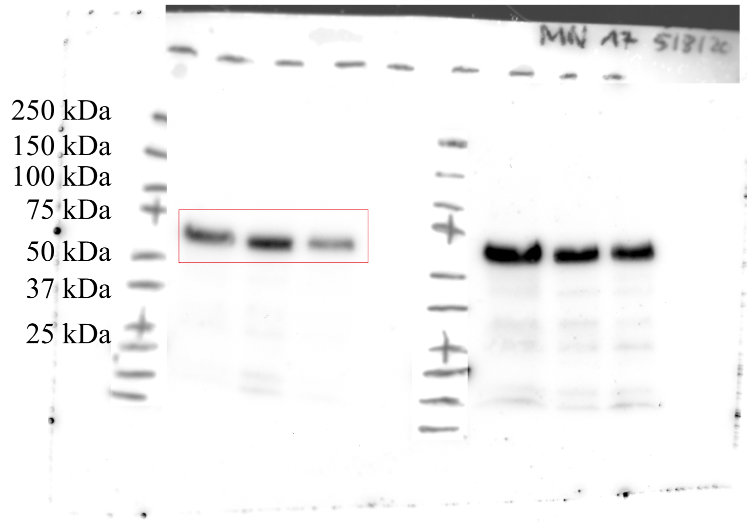

Figure 3A (left), actin

actin (approx. 45 kDa)

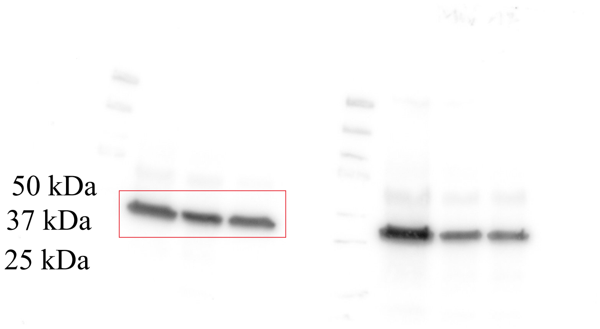

Supplementary Fig S17 : Unprocessed replicate blots of Figure 3A (left)

Supplementary Figure S18

n2

Figure 3A (right), AMPK

AMPK (approx. 62 kDa)

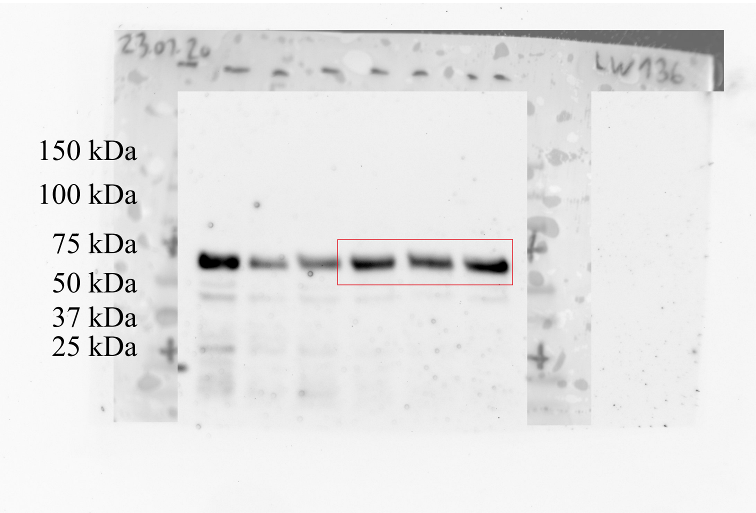

Figure 3A (right), actin

actin (approx. 45 kDa)

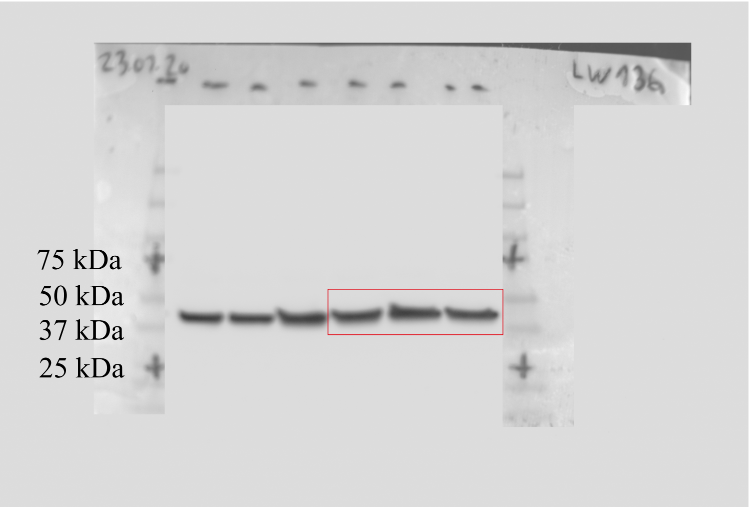

n3

Figure 3A (right), AMPK

AMPK (approx. 62 kDa)

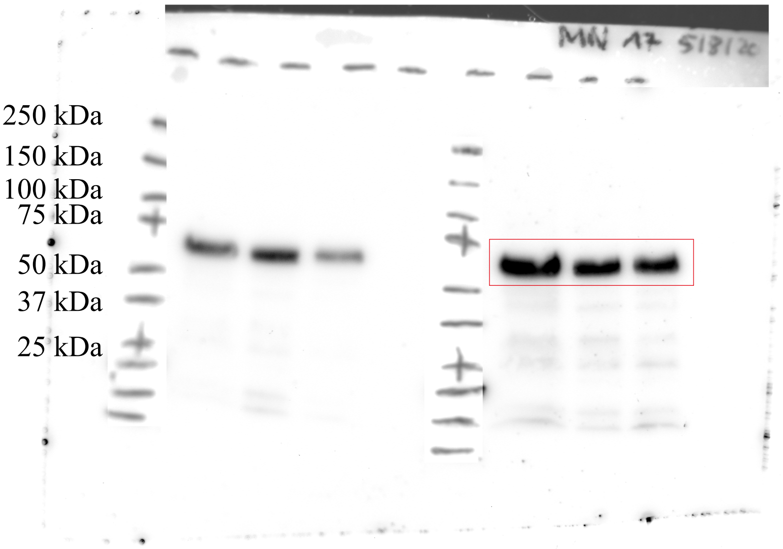

Figure 3A (right), actin

actin (approx. 45 kDa)

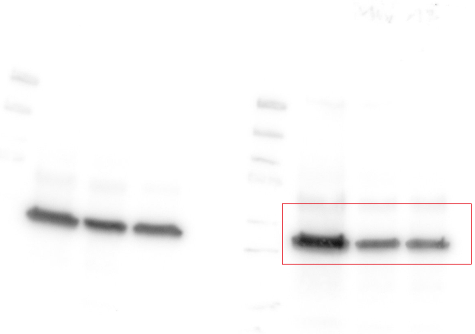

Supplementary Fig S18 : Unprocessed replicate blots of Figure 3A (right)

Supplementary Figure S19

n2

Figure 3B (left), pAMPK

pAMPK (approx. 62 kDa)

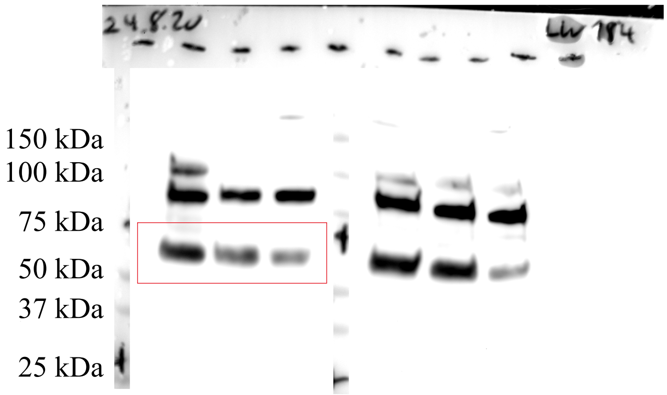

Figure 3B (left), actin

actin (approx. 45 kDa)

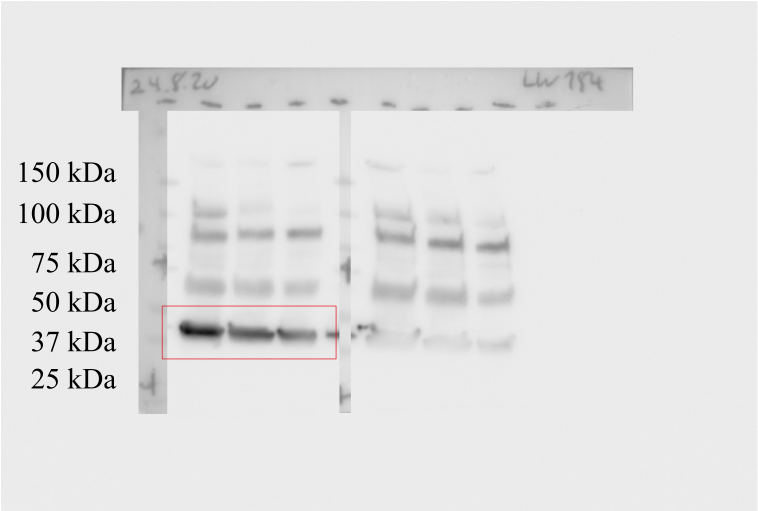

n3

Figure 3B (left), pAMPK

pAMPK (approx. 62 kDa)

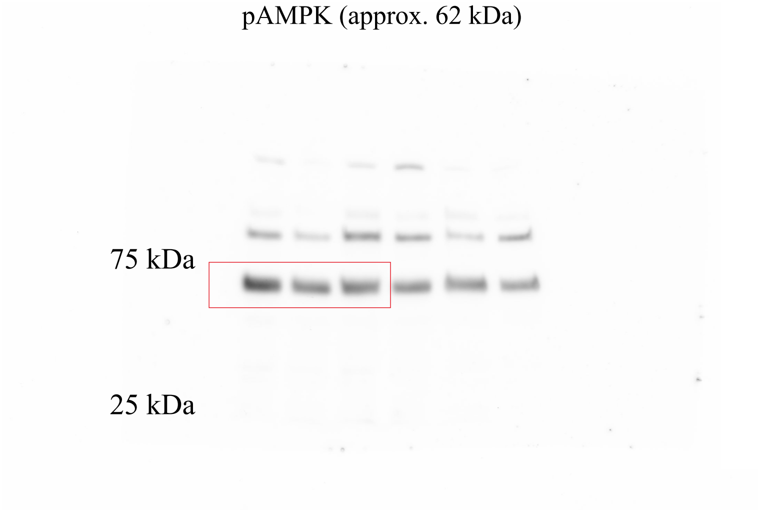

Figure 3B (left), actin

actin (approx. 45 kDa)

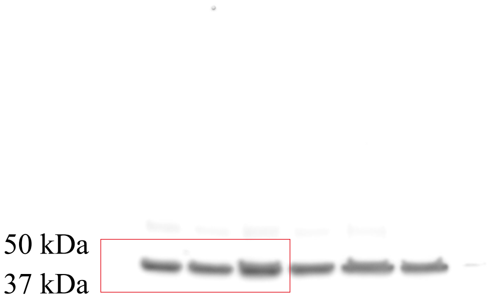

Supplementary Fig S19 : Unprocessed replicate blots of Figure 3B (left)

Supplementary Figure S20

n2

Figure 3B (right), pAMPK

pAMPK (approx. 62 kDa)

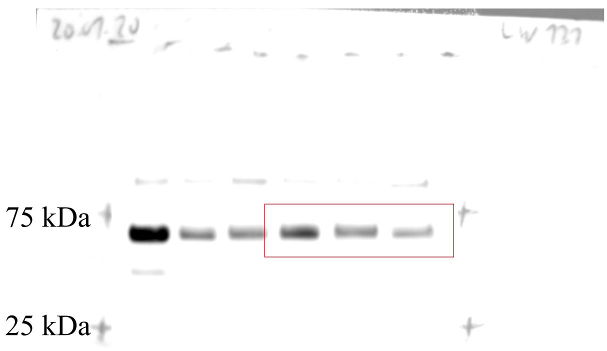

Figure 3B (right), actin

actin (approx. 45 kDa)

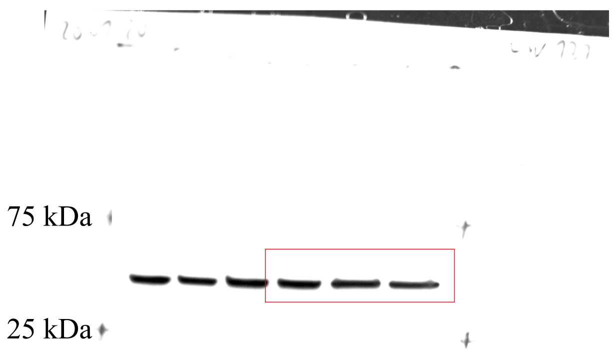

n3

Figure 3B (right), pAMPK

pAMPK (approx. 62 kDa)

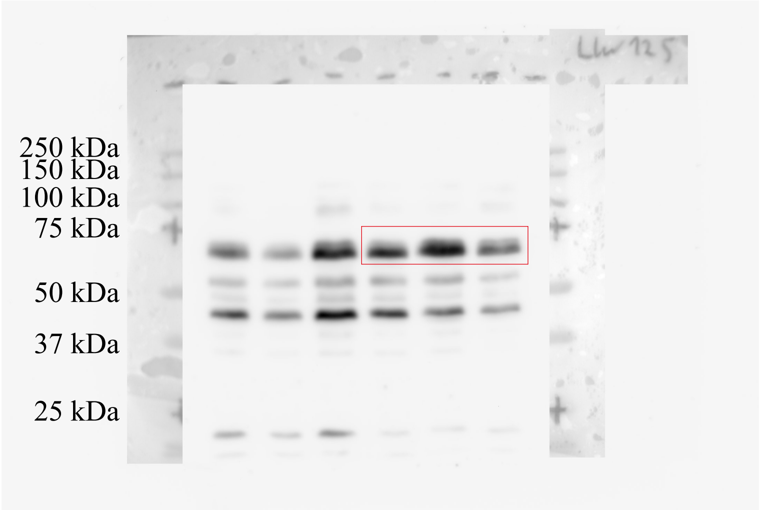

Figure 3B (right), actin

actin (approx. 45 kDa)

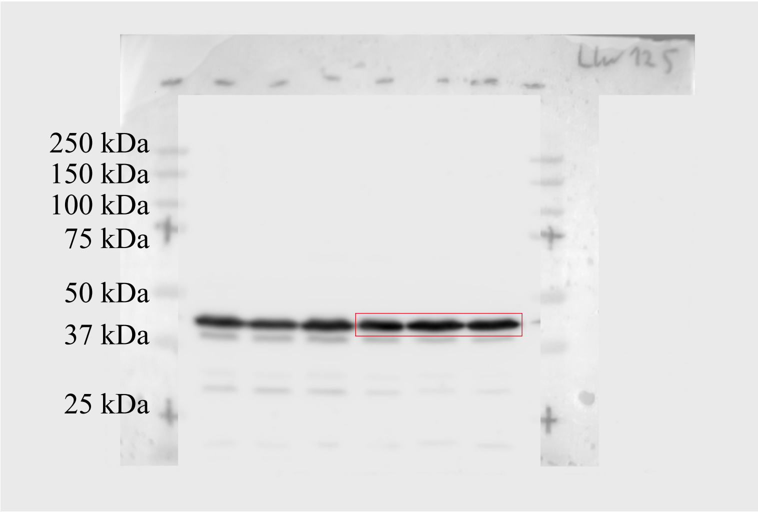

Supplementary Fig S20 : Unprocessed replicate blots of Figure 3B (right)

Supplementary Figure S21

n2

Figure 3C (left), CaMKII

CaMKII (approx. 50 kDa)

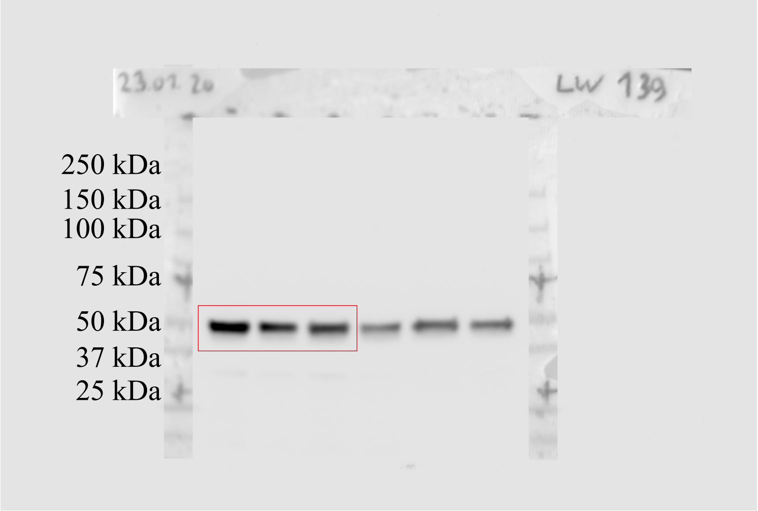

Figure 3C (left), actin

actin (approx. 45 kDa)

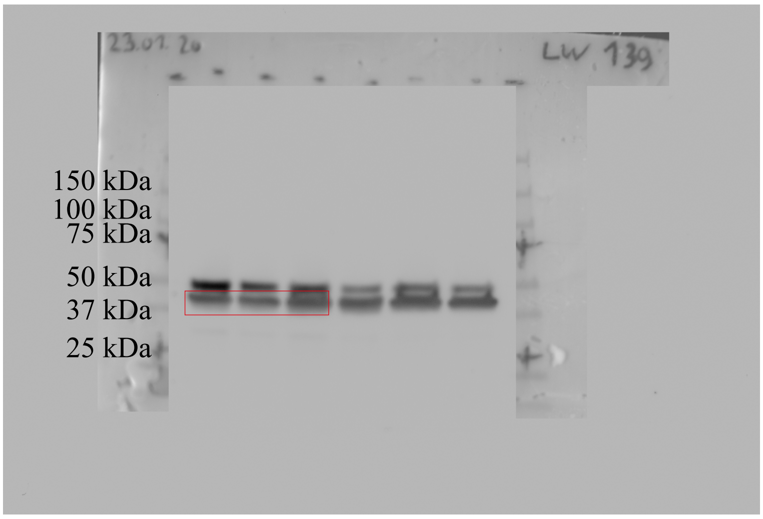

n3

Figure 3C (left), CaMKII

CaMKII (approx. 50 kDa)

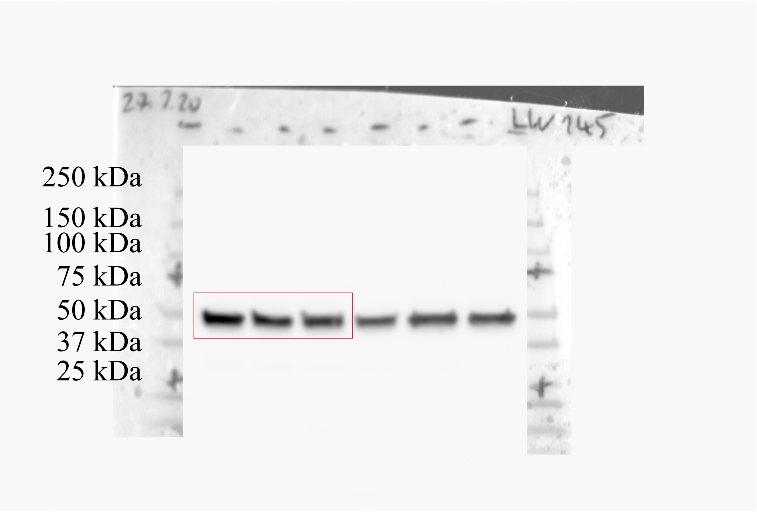

Figure 3C (left), actin

actin (approx. 45 kDa)

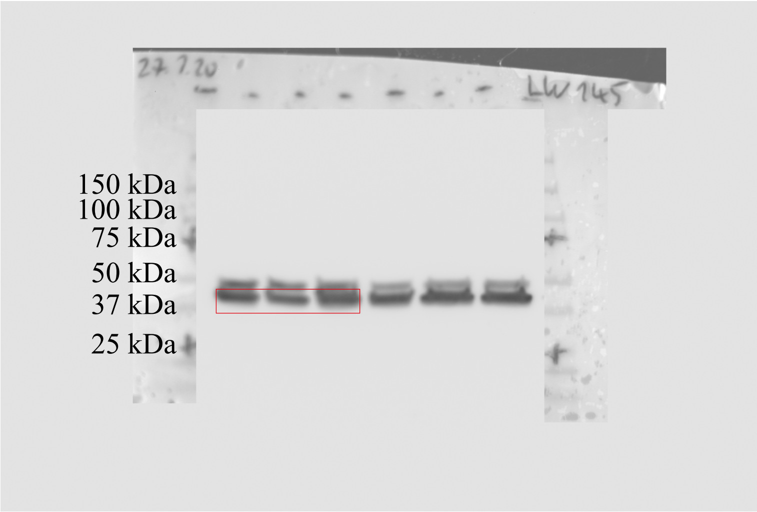

Supplementary Fig S21 : Unprocessed replicate blots of Figure 3C (left)

Supplementary Figure S22

n2

Figure 3C (right), CaMKII

CaMKII (approx. 50 kDa)

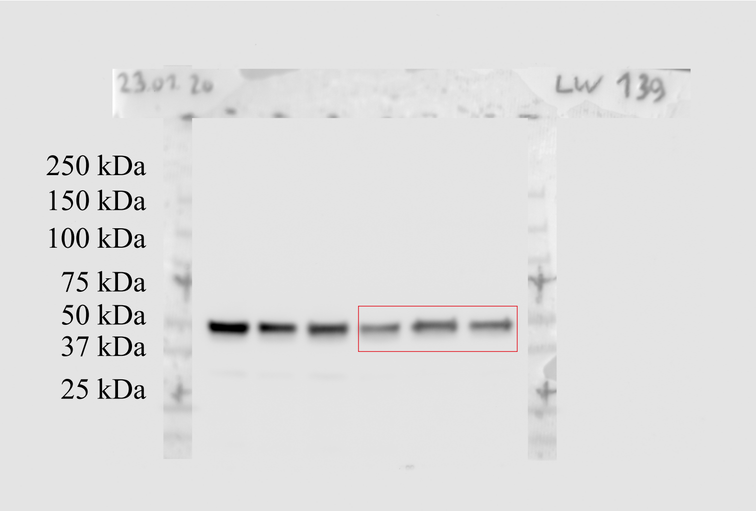

Figure 3C (right), actin

actin (approx. 45 kDa)

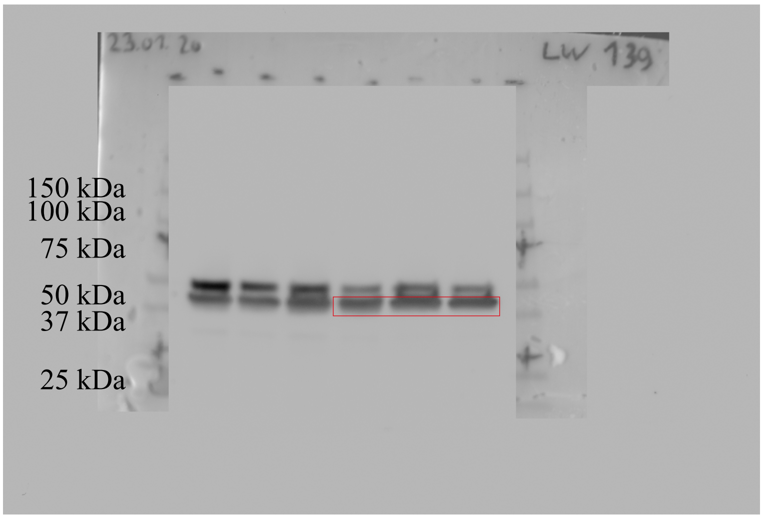

n3

Figure 3C (right), CaMKII

CaMKII (approx. 50 kDa)

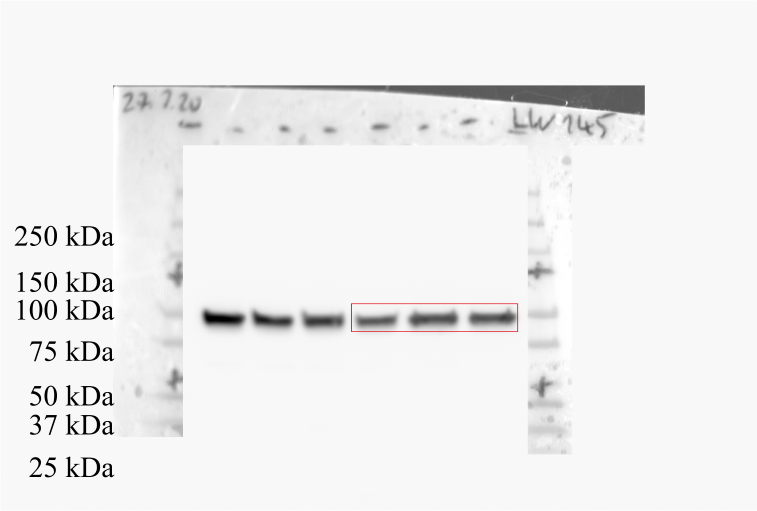

Figure 3C (right), actin

actin (approx. 45 kDa)

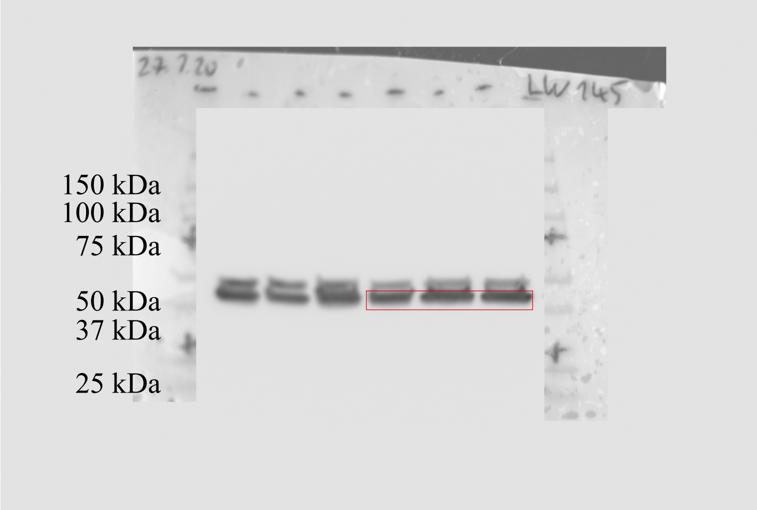

Supplementary Fig S22 : Unprocessed replicate blots of Figure 3C (right)

Supplementary Figure S23

n2

Figure 3D, YAP

YAP (approx. 70 kDa)

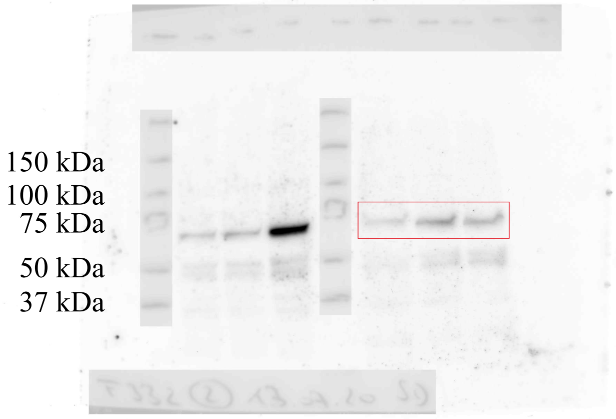

Figure 3D, actin

actin (approx. 45 kDa)

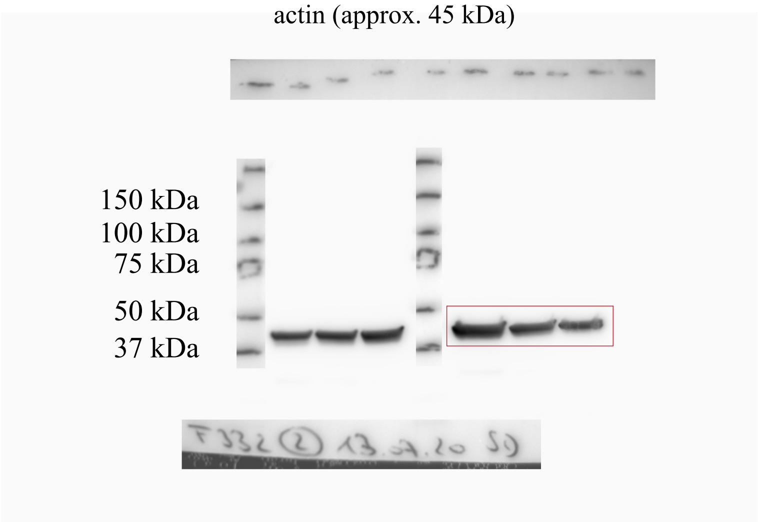

n3

Figure 3D, YAP

YAP (approx. 70 kDa)

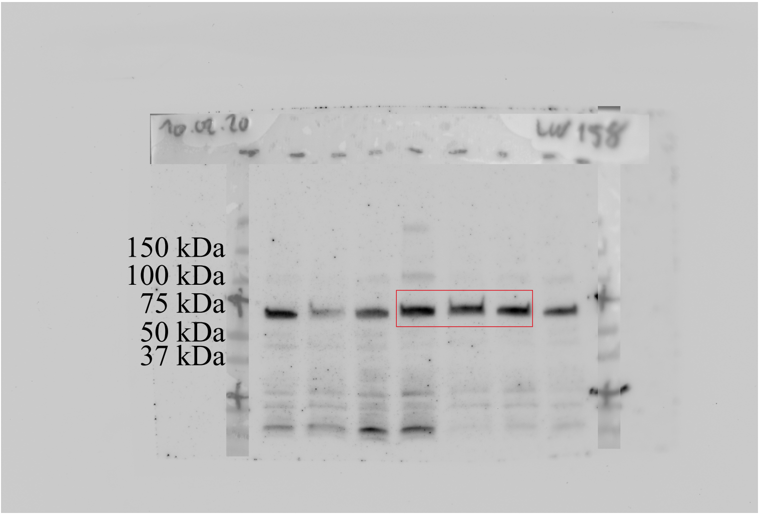

Figure 3D, actin

actin (approx. 45 kDa)

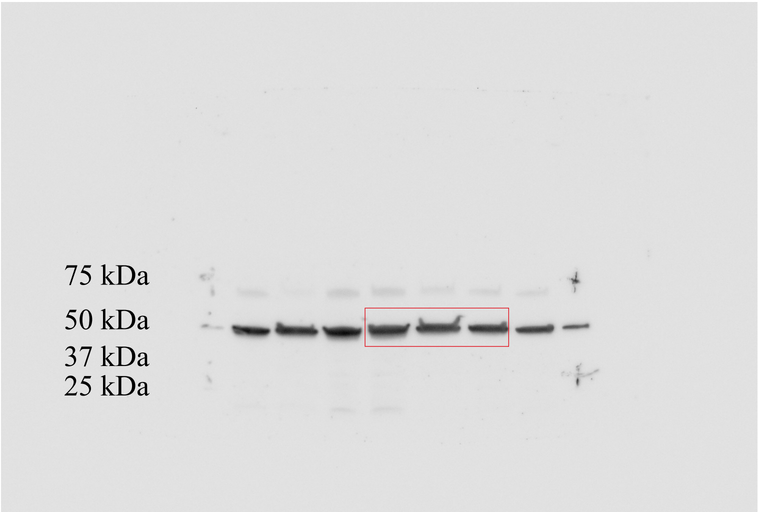

Supplementary Fig S23 : Unprocessed replicate blots of Figure 3D (right)

Supplementary Figure S24

n2

Supplementary Figure 1A, pPERK

pPERK (approx. 170 kDa)

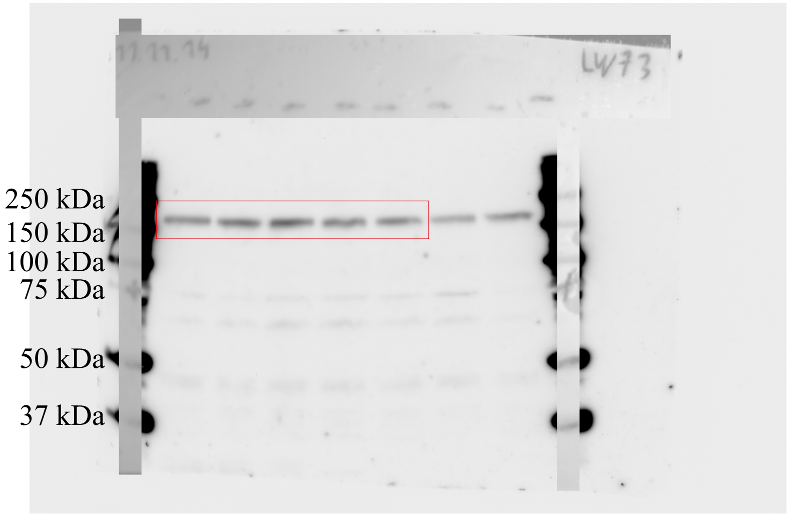

Supplementary Figure 1A, actin

actin (approx. 45 kDa)

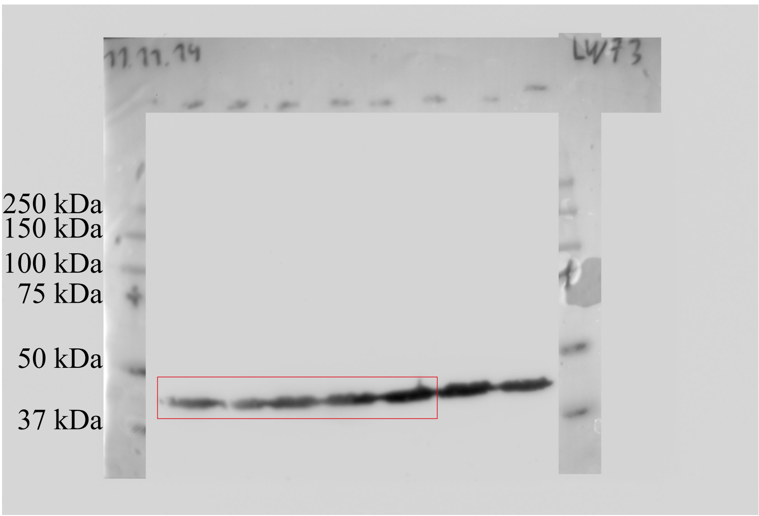

n3

Supplementary Figure 1A, pPERK

pPERK (approx. 170 kDa)

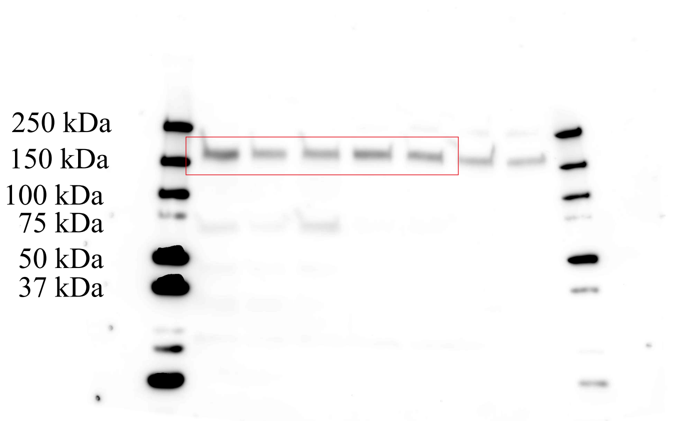

Supplementary Figure 1A, actin

actin (approx. 45 kDa)

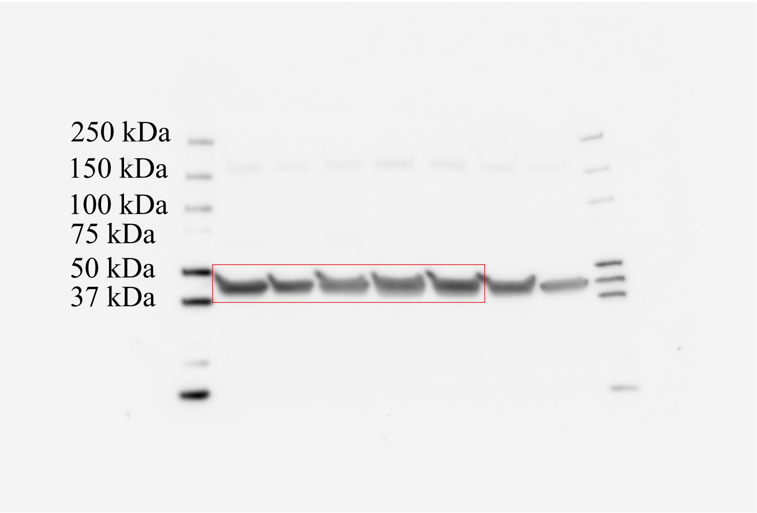

Supplementary Fig S24 : Unprocessed replicate blots of Supplementary Figure S1 A

Supplementary Figure S25

n2

Supplementary Figure 1B, CaMKII

CaMKII (approx. 50 kDa)

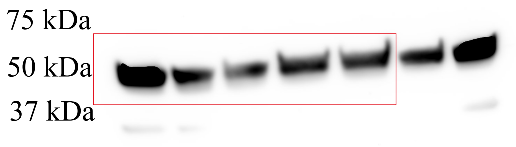

Supplementary Figure 1B, actin

actin (approx. 45 kDa)

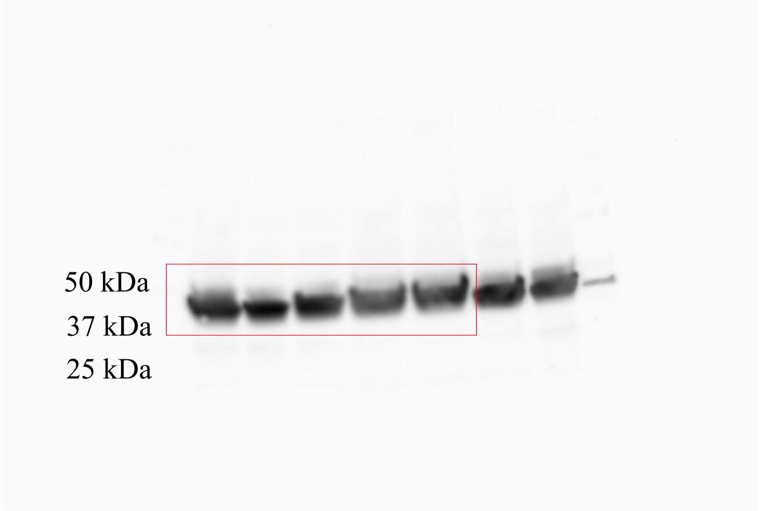

n3

Supplementary Figure 1B, CaMKII

CaMKII (approx. 50 kDa)

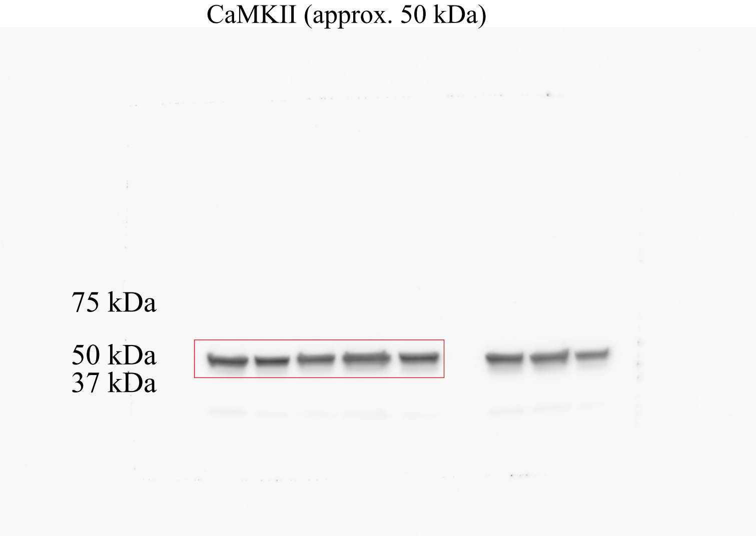

Supplementary Figure 1B, actin

actin (approx. 45 kDa)

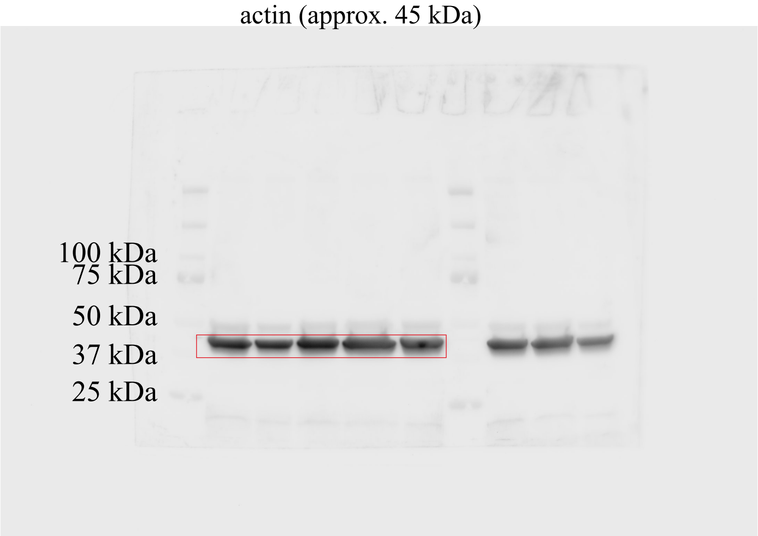

Supplementary Fig S25 : Unprocessed replicate blots of Supplementary Figure S1 B

Supplementary Figure S26

n2

Supplementary Figure 1C
pAMPK (approx. 62 kDa)

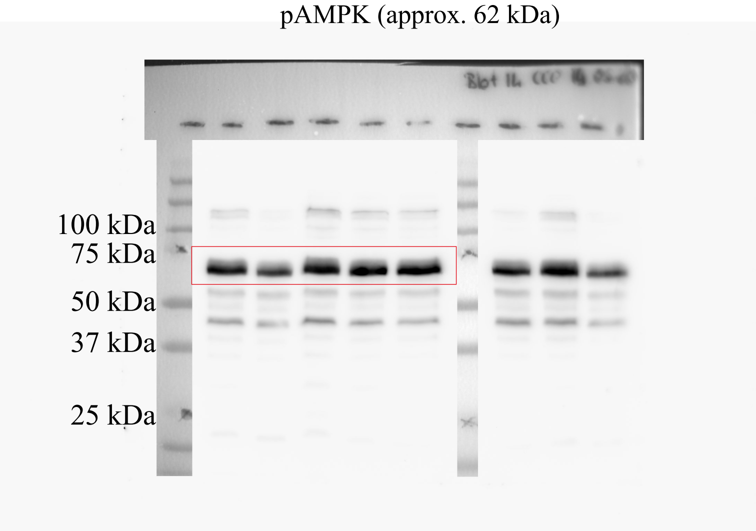

Supplementary Figure 1C, actin
actin (approx. 45 kDa)

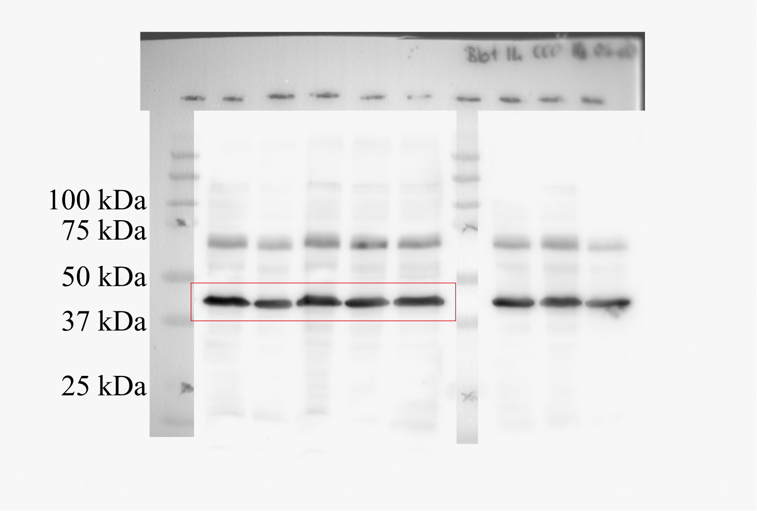

n3

Supplementary Figure 1C
pAMPK (approx. 62 kDa)

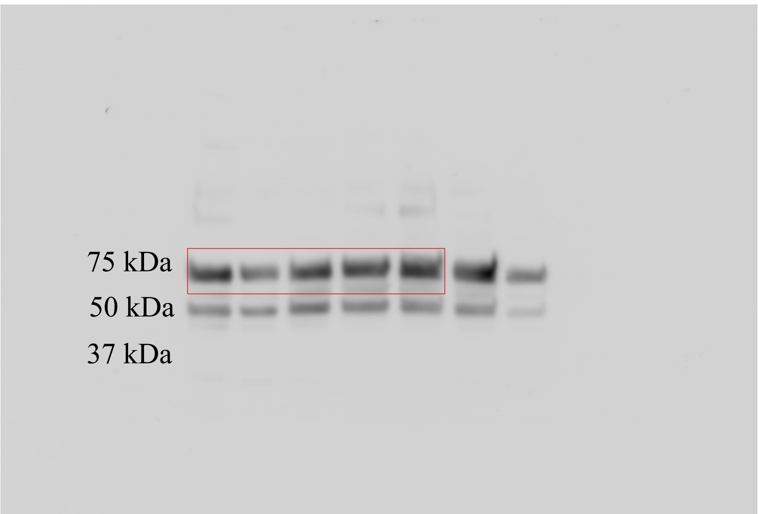

Supplementary Figure 1C, actin
actin (approx. 45 kDa)

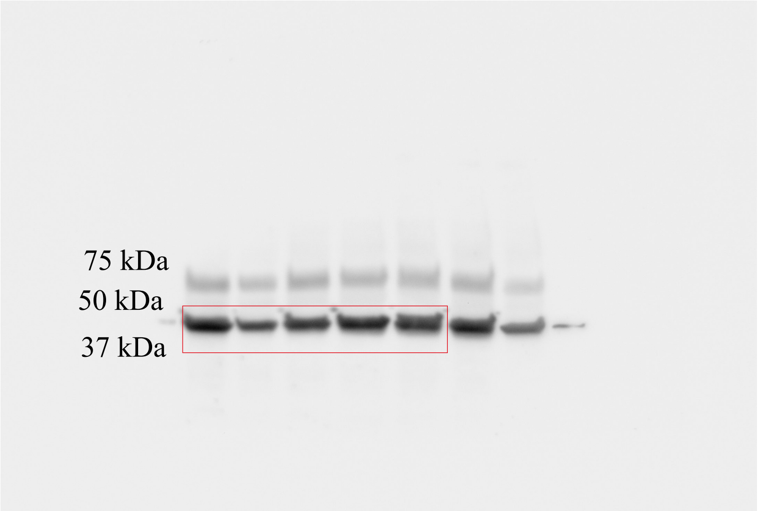

Supplementary Fig S26 : Unprocessed replicate blots of Supplementary Figure S1 C
