# Supplementary material for: Wildtype heterogeneity contributes to clonal variability in genome edited cells
Source: Sci Rep. 2022 Oct 28;12:18211. doi: 10.1038/s41598-022-22885-8 (PMC9616811; doi:10.1038/s41598-022-22885-8)
Supplement: Supplementary file 2 — Supplementary Information 2. [file 41598_2022_22885_MOESM2_ESM.docx]

**Supplementary Data Legends**

**Supplementary Data 1:** Differential gene expression data for comparison between monoclonal WT5 and polyclonal wild-type cell line as depicted in Fig. 4.

**Supplementary Data 2:** Differential gene expression data for comparison between monoclonal WT5 and corresponding subclones as depicted in Fig. 5.
